# Genome-Wide Identification and Characterization of the Brassinazole-resistant (*BZR*) Gene Family and Its Expression in the Various Developmental Stage and Stress Conditions in Wheat (*Triticum aestivum* L.)

**DOI:** 10.3390/ijms22168743

**Published:** 2021-08-14

**Authors:** Mahipal Singh Kesawat, Bhagwat Singh Kherawat, Anupama Singh, Prajjal Dey, Mandakini Kabi, Debanjana Debnath, Debanjana Saha, Ansuman Khandual, Sandeep Rout, Manorama, Asjad Ali, Ramasubba Reddy Palem, Ravi Gupta, Avinash Ashok Kadam, Hyun-Uk Kim, Sang-Min Chung, Manu Kumar

**Affiliations:** 1 Institute for Molecular Biology and Genetics, School of Biological Sciences, Seoul National University, Seoul 08826, Korea; mahipal.s@srisriuniversity.edu.in; 2 Faculty of Agriculture, Sri Sri University, Cuttack 754-006, India; anupama.s@srisriuniversity.edu.in (A.S.); prajjal.d@srisriuniversity.edu.in (P.D.); mandakini.k@srisriuniversity.edu.in (M.K.); debanjana.d@srisriuniversity.edu.in (D.D.); ansuman.k@srisriuniversity.edu.in (A.K.); sandeep.r@srisriuniversity.edu.in (S.R.); 3 Krishi Vigyan Kendra, Bikaner II, Swami Keshwanand Rajasthan Agricultural University, Bikaner 334603, India; skherawat@gmail.com; 4 Department of Biotechnology, Centurion University of Technology and Management, Bhubaneshwar 752050, India; debanjana.saha@cutm.ac.in; 5 Department of Dairy Microbiology, College of Dairy Science and Food Technology, Raipur 49200, India; manoramachauhan2@gmail.com; 6 Department of Agriculture and Fisheries, Mareeba, QLD 4880, Australia; mirza_asjad@hotmail.com; 7 Department of Medical Biotechnology, Biomedical Campus, Dongguk University, Seoul 10326, Korea; palemsubbareddy@gmail.com; 8 Department of Botany, School of Chemical and Life Sciences, Jamia Hamdard, New Delhi 110062, India; dr.ravigupta@jamiahamdard.ac.in; 9 Research Institute of Biotechnology and Medical Converged Science, Dongguk University-Seoul, 32 Dongguk-ro, Ilsandong-gu, Goyang 10326, Korea; kadamavinash@dongguk.edu; 10 Department of Bioindustry and Bioresource Engineering, Plant Engineering Research Institute, Sejong University, Seoul 05006, Korea; hukim64@sejong.ac.kr; 11 Department of Life Science, College of Life Science and Biotechnology, Dongguk University, Goyang 10326, Korea; smchung@dongguk.edu

**Keywords:** brassinosteroid, qRT-PCR, cis-acting regulatory elements, biotic and abiotic stress

## Abstract

Brassinosteroids (BRs) play crucial roles in various biological processes, including plant developmental processes and response to diverse biotic and abiotic stresses. However, no information is currently available about this gene family in wheat (*Triticum aestivum* L.). In the present investigation, we identified the *BZR* gene family in wheat to understand the evolution and their role in diverse developmental processes and under different stress conditions. In this study, we performed the genome-wide analysis of the *BZR* gene family in the bread wheat and identified 20 *TaBZR* genes through a homology search and further characterized them to understand their structure, function, and distribution across various tissues. Phylogenetic analyses lead to the classification of *TaBZR* genes into five different groups or subfamilies, providing evidence of evolutionary relationship with *Arabidopsis thaliana*, *Zea mays*, *Glycine max*, and *Oryza sativa*. A gene exon/intron structure analysis showed a distinct evolutionary path and predicted the possible gene duplication events. Further, the physical and biochemical properties, conserved motifs, chromosomal, subcellular localization, and cis-acting regulatory elements were also examined using various computational approaches. In addition, an analysis of public RNA-seq data also shows that *TaBZR* genes may be involved in diverse developmental processes and stress tolerance mechanisms. Moreover, qRT-PCR results also showed similar expression with slight variation. Collectively, these results suggest that *TaBZR* genes might play an important role in plant developmental processes and various stress conditions. Therefore, this work provides valuable information for further elucidate the precise role of BZR family members in wheat.

## 1. Introduction

The integration of phytohormone has regulated plant developmental processes; however, their intricate regulatory network is often influenced by diverse internal and external stimuli such as biotic and abiotic stresses [1,2,3,4,5,6]. Several genes, especially transcription factors, tend to coordinate with various hormone signaling to respond to endogenous and external stimuli to adapt to the adverse conditions and maintain plant growth and development [2,7,8,9,10]. Brassinosteroid (BR) plays a vital role in numerous physiological processes, including cell division and elongation, vascular differentiation, seed germination, flowering, stomata formation, pollen tube growth, and senescence, xylem cell differentiation, and photomorphogenesis [3,11,12,13,14,15,16]. Moreover, BR also plays a crucial role in plant responses to diverse biotic and abiotic stresses, such as insects, fungal, bacterial, viral diseases, heat, drought, cold, salinity, and UV radiation [17,18,19,20,21,22]. BR signaling pathway has been extensively studied using genetic, molecular, and proteomic approaches over the past decade. It gives a global view of the major component and the molecular mechanism of BR signaling [11,12,13,15]. Briefly, an extracellular receptor kinase (LRRRK) Brassinosteroid-Insensitive 1 (BRI1) and BRI1-ASSOCIATED RECEPTOR KINASE 1 (BAK1) can be perceived and bind to BR, resulting in the initiation of BR signaling [23,24]. A series of phosphorylation and dephosphorylation activates the two homologous transcription factors, BRASSINAZOLE-RESISTANT 1 (BZR1) and BRI1-EMS SUPPRESSOR 1 (BES1) [23,25,26,27]. The activation of the BR signal, the BZR1/BES1 transcription factors bind to the BR response element (CGTGT/CG) or E-box (CANNTG) to regulate the expression of thousand target genes [28,29,30]. Thus, BZR1 and BES1 are considered the main transcription factors in BR signaling. Furthermore, the BZR signaling components are highly conserved from primitive to modern plants [25,31,32,33]. Although the sizes of their genomes and their numbers of genes differ significantly in plants, for instance, 6 BZR genes in *Arabidopsis thaliana*, 11 in *Zea mays*, 6 in *Beta vulgaris*, 9 in *Solanum lycopersicum*, 6 in *Cajanus cajan*, 16 in *Glycine max*, 7 in *Medicago truncatula*, 6 in *Cicer arietinum*, and 15 in *Brassica rapa*, respectively [31,32,33,34,35,36].

BZR proteins have been well characterized in the model plant Arabidopsis, which includes six members (AtBZR1; AtBES1 and 4 BES1 homologs: AtBEH1–4) that are highly conserved in sequence and functional domain structure [24,37]. In Arabidopsis, a single mutant of AtBZR1, AtBES1, and four homologs AtBEH1–4 did not exhibit any phenotype. Furthermore, no growth defects were observed in different combinations of double, triple, and quadruple mutants [37]. However, tapetum and microsporocyte development defects in anthers were observed in the quintuple mutant (*bes1*/*bzr1*/*beh1*/*beh3*/*beh4*) and sextuple mutant (*bes1*/*bzr1*/*beh1*/*beh2*/*beh3*/*beh4*), suggesting the highly functional redundancy of BES1 genes [37,38]. The gain-of-function mutants of *Atbzr1-1D* displayed shorter petioles and hypocotyls, delayed flowering, and produced more leaves than wild-type [25]. Overexpression of the AtDWF4 in *Brassica napus* enhanced root length, higher root biomass, seed yield, and tolerance to biotic and abiotic stress [39]. BRI1 gene independently regulates another development in *Arabidopsis thaliana*. In addition, BZR family members have also shown function in responses to diverse environmental stress in *Arabidopsis thaliana* [28,40,41]. However, little is known about BZRs functions in other plant species; for instance, overexpression of GmBZL2 enhanced BR signaling, resulting in increased seed number per silique, while mutant lines exhibited curly leaves and delayed flowering in *Arabidopsis thaliana* [42]. The *BrBZR* gene family has been regulating diverse biotic and abiotic stress-related pathways [43]. Overexpression *brassinazole-resistant 2* (TaBZR2) showed drought tolerance, while suppression of TaBZR2 by RNA interference resulted in elevated drought sensitivity in wheat [44]. Heterologous expression of ZmBES1/BZR1-5 exhibited shoot growth and root development and enhanced osmotic and salt tolerance in *Arabidopsis thaliana* [45]. OsBZR1 positively regulates BR signaling pathways in the rice, while 14-3-3 proteins inhibit OsBZR1 accumulation in the nucleus to negatively regulate BR signaling [46]. GmBEHL1 facilitates the crosstalk between nodulation signaling and BR signaling pathways that negatively regulate nodulation in soybean [47].

With recent advances in sequencing technologies, there has been a dramatic increase in the number of plants sequenced genomes in the past years. Although, genome sequence databases have provided researchers with a wealth of encoded information. However, the gene identified in the plant species genome is still uncharacterized, particularly in their function and regulation. Therefore, the structural and functional characterization of those genes is now a challenging approach [48,49]. Bread wheat (*Triticum aestivum* L.) is a significant cereal and staple crop worldwide, providing food for humans and feed for animals [50,51]. Wheat is an essential source of carbohydrates, protein, vitamins, and minerals for humans [51,52,53,54]. However, wheat production is adversely affected by several biotic and abiotic stresses such as insects, fungal, bacterial, viral diseases, heat, drought, cold, and salinity [55,56]. Several researchers have been concentrated on improving productivity, quality and stress tolerance in wheat. Therefore, this work design to understand the structure, function, evolution of BZR gene family and their role in diverse developmental processes and under different stress conditions. However, the genome-wide analysis of the BZR gene family in wheat has yet to be investigated. In the present investigation, a comprehensive analysis of the wheat BZR gene family was carried out using various computational approaches. We analyzed the genomic localization, chromosomal distribution, exon-intron structure, conserved motif, cis-elements, phylogenetic and synteny relationships. In addition, we also examined the expression patterns of TaBZR genes in various tissue and diverse biotic and abiotic stress conditions. Thus, the outcome of this study will be helpful to understand the role of BZRs in plant developmental processes and various stress conditions and their sequential execution to increase yield and develop the stress-tolerant variety in the wheat.

## 2. Results

### 2.1. Identification of BZR Family Members in T. aestivum

A total of 20 BZR genes were identified in the wheat genome (Table 1); this number is relatively higher than the previously reported BZRs in different plant species such as Arabidopsis, rice, cotton sorghum, and maize (Table 2).

The protein characteristics of the TaBZR, such as coding sequence (CDS) lengths, protein lengths, isoelectric point, and molecular weight, were evaluated, listed in Table 1. CDS length ranged from 537 (*TaBZR2.5*) to 2061 bp (*TaBZR3.2*). The TaBZR family had a molecular weight range from 19.26 to 75.48 kDa. TaBZR2.5 had the lowest molecular weight, 19.26, whereas TaBZR4.3 had the highest molecular weight, 75.48. The average isoelectric point (pI) of the TaBZR gene family ranged from 5.2 and 9.1; TaBZR2.5 had the highest pI, 9.1, while TaBZR3.1 and TaBZR3.2 had the lowest pI 5.2. We also plotted the molecular weight of TaBZR with their pI to examine the molecular weight distribution of different TaBZR family members (Figure S1). The plots indicate that few TaBZRs have similar molecular weight, pI, and are clustered together. The calculated grand average of hydropathy index (GRAVY) values of TaBZRs were −0.3483 to −0.7511, indicated that they were hydrophilic in nature. The determination of the subcellular localization of TaBZR proteins will help understand the molecular function. A subcellular localization prediction of TaBZR proteins suggesting that all TaBZRs were located on the nucleus (Table 1).

**Figure 1:**
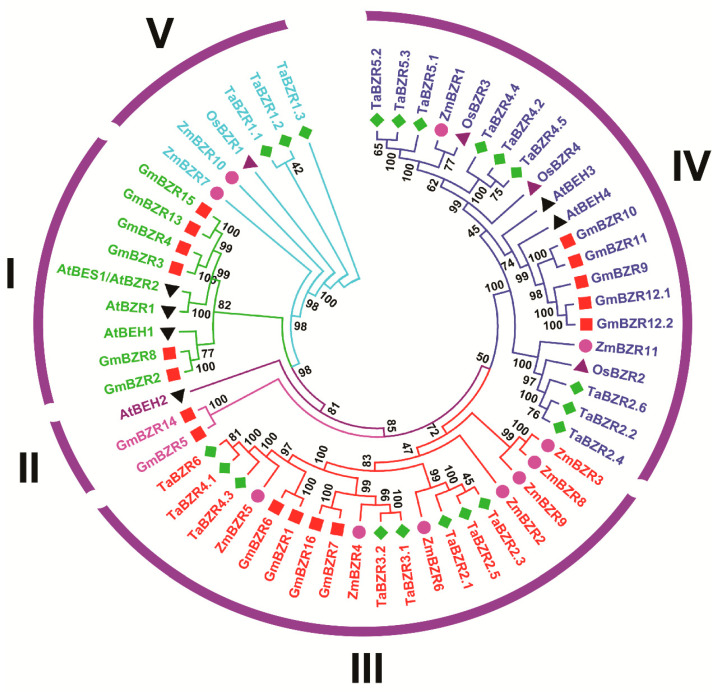
Phylogenetic analysis of BZR proteins among the wheat (20), Arabidopsis (6), maize (11), soybean (16), and rice (4) using the MEGAX by the neighbor-joining method.

To understand the evolutionary relationship between TaBZRs and other plant species BZRs, the phylogenetic tree was constructed with MEGA version 10 by comparing 6 AtBZRs, 11 ZmBZRs, 4 OsBZRs, and 16 GmBZRs proteins (Table S2). The phylogenetic results revealed that BZR proteins were divided into five groups or subfamilies (Figure 1). Group IV was the largest, with nine TaBZR members. None of the TaBZR members were found in groups I and II. Groups III and V included eight and three members, respectively (Figure 1 and Figure S2).

### 2.2. Chromosomal Distribution of TaBZR Genes

**Figure 2:**
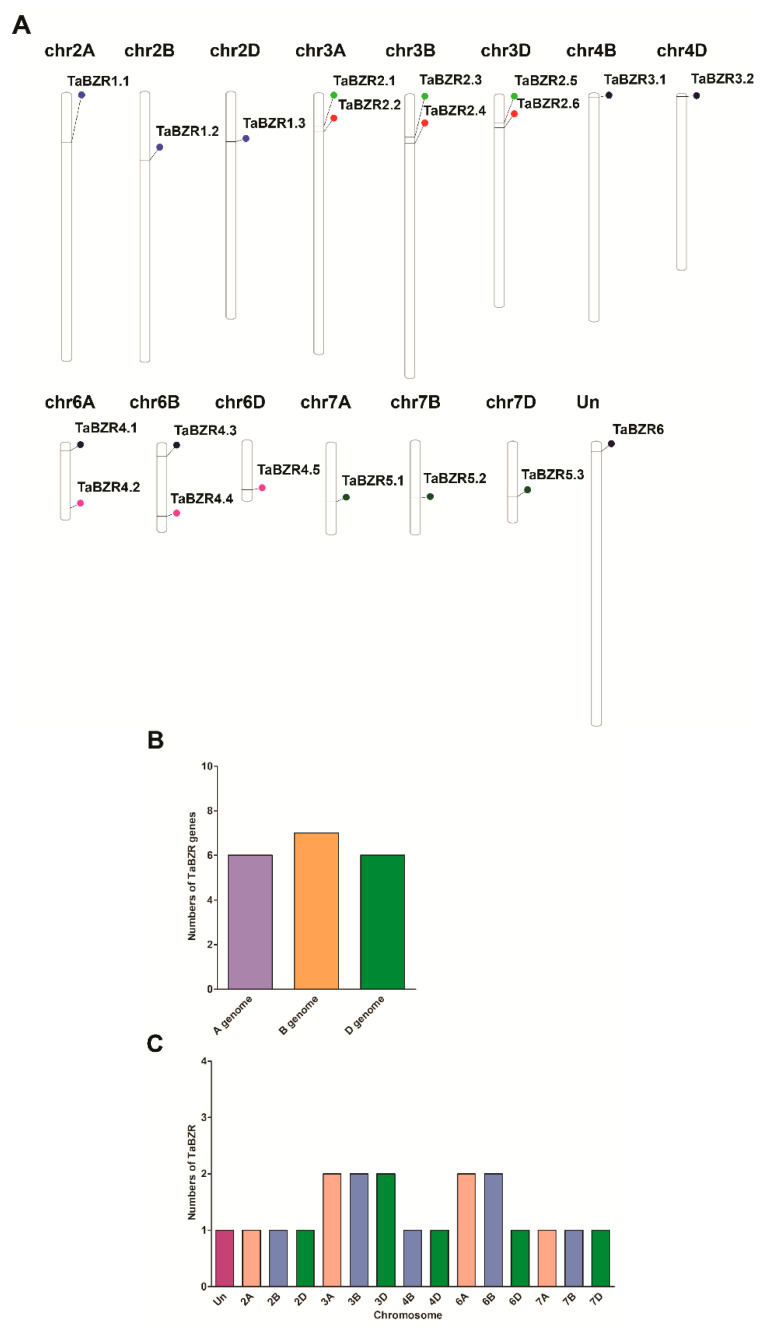
Genomic distribution of identified BZR genes on the 21 chromosomes of wheat and within the 3 subgenomes. (**A**) Schematic representations of the chromosomal distribution of BZR genes on the 21 chromosomes of wheat and the name of the gene on the right side. The colored circle on the chromosomes indicates the position of the BZR genes. The chromosome numbers of the three subgenomes are indicated at the top of each bar. (**B**) Distribution of BZR genes in the three subgenomes. (**C**) Distribution of BZR genes across 21 chromosomes.

Genomic chromosomal distribution of the identified TaBZR genes in wheat was mapped to the corresponding chromosomes according to the chromosomal locations of BZR genes by the PhenGram online server. The TaBZR genes are present on the 15 wheat chromosomes (Figure 2A and Table 1). TaBZR genes displayed a slightly higher presence on B subgenomes (Figure 2B). Maximum TaBZR genes, for instance, seven were mapped on the chromosomes of the B subgenome. A and D subgenome had the least number of TaBZR genes, i.e., six. The maximum numbers of TaBZRs are located on chromosomes 3A, 3B, 3D, 6A, and 6B with two genes (Figure 2C). The minimum number of TaBZRs are located on chromosomes 2A, 2B, 2D, 4B, 4D, 6D, 7A, 7B, and 7D, having the only single gene, respectively. Conversely, none of the TaBZR genes were found on chromosomes 1 and 5. Overall, all the BZR family genes were evenly distributed on the three subgenome of wheat.

To investigate in the context that the wheat is hexapolyploid with large genomes, we further examine the duplication events in the TaBZR gene family. The phylogenetic analysis of the TaBZR genes also indicates several duplication events (Figure S3). We found 14 BZR genes in *T. aestivum* that participated in duplication events (Figure S4 and Table S3), which point out that the expansion of the BZR gene family in *T. aestivum* was caused mainly by whole-genome duplication or segmental duplication within genomes. To elucidate the selective pressure on the duplicated TaBZR genes, we calculated the non-synonymous (Ka), synonymous substitution (Ks), and the Ka/Ks ratios for the seven TaBZR gene pairs (Table S3). The value of Ka/Ks = 1 denotes that genes experienced a neutral selection; <1 suggests a purifying or negative selection, and >1 indicates a positive selection [57]. The Ka/Ks values for seven gene pairs were less than 1, suggesting that TaBZR genes underwent a strong purifying or negative selection pressure with slight changes after duplication. Thus, these results indicating the conserved evolution of TaBZR genes.

**Figure 3:**
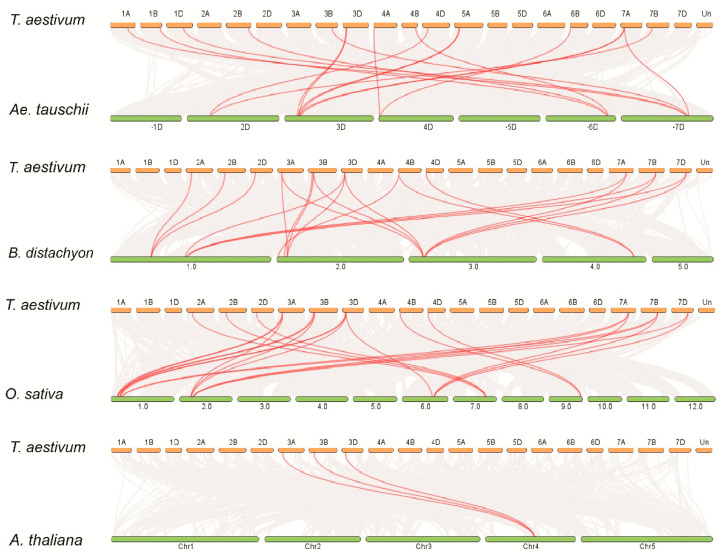
Syntenic relationships of TaBZR genes between *Aegilops tauschii*, *Brachypodium distachyon*, *Oryza sativa* and *Arabidopsis thaliana*. The gray lines in the background represent the collinear blocks within *Triticum aestivum* and other plant genomes, while the red lines highlight the syntenic BZR gene pairs.

To further examine the synteny relationships of TaBZR genes with other wheat relatives and model plants such as *B. distachyon*, *Ae. tauschii*, *T. dicoccoides*, *O. sativa*, and *A. thaliana*, a Multiple Collinearity Scan toolkit was used to find the orthologous genes between these plant species genomes (Figure 3 and Table S4).

A total of 17, 42, 20, and 26 orthologous gene pairs between TaBZRs with other BZR genes in *B. distachyon*, *Ae. tauschii*, *T. dicoccoides*, *O. sativa*, and *A. thaliana*, respectively. A few TaBZR genes had at least three pairs of orthologous genes, for instance, TaBZR1.1, TaBZR1.2, and TaBZR1.3, which might have played a crucial role in the evolution of BZR genes. These results suggested that TaBZR genes in wheat might be originated from other plant species orthologous genes.

### 2.3. Gene Structure and Conserved Motif Analysis of TaBZR Genes

**Figure 4:**
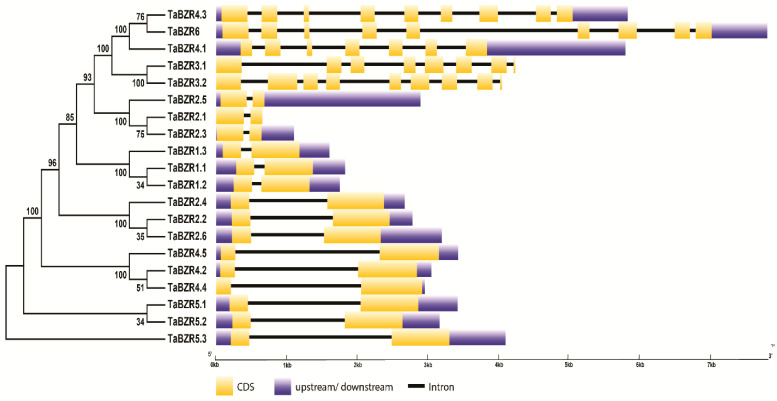
Exon-intron organization of the TaBZR genes. Yellow boxes represent exons, untranslated regions (UTRs) are indicated by blue boxes, and black lines represent introns. The lengths of the boxes and lines are scaled based on gene length. The exon and intron sizes can be estimated using the scale at the bottom.

**Figure 5:**
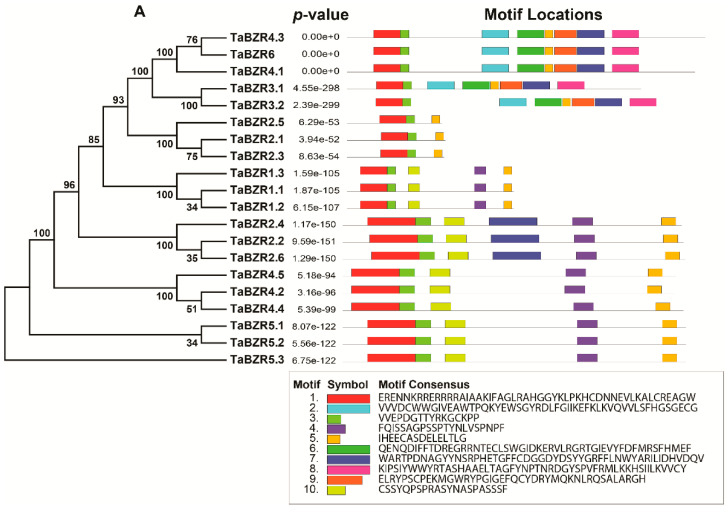

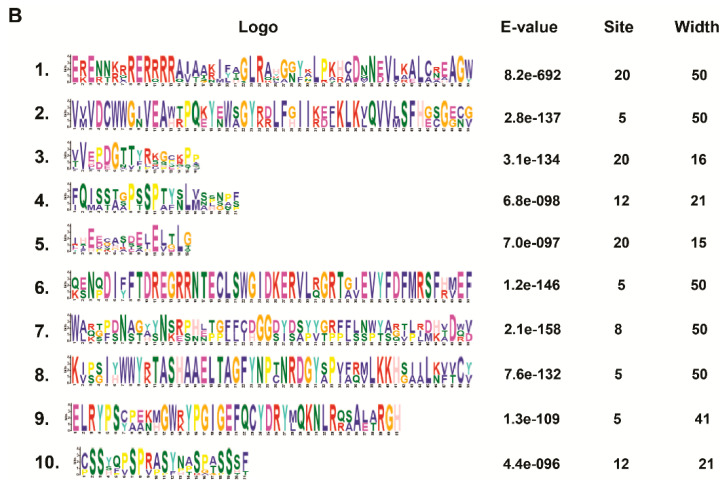
Conserved motifs of TaBZR genes elucidated by MEME. (**A**) Colored boxes representing different conserved motifs having different sequences and sizes. (**B**) Sequence logo conserved motif of the wheat BZR proteins. The overall height of each stack represents the degree of conservation at this position, while the height of the individual letters within each stack indicates the relative frequency of the corresponding amino acids.

To understand the structural characteristics of the TaBZR genes, the exon-intron structures (Figure 4) and conserved motifs (Figure 5A,B) of TaBZR genes were analyzed. Gene structure analysis revealed that the TaBZR gene family varied greatly in terms of gene structure as most of BZR genes contain two exons, while most of the TaBZR genes are had one intron. Maximum eight introns were found in the *TaBZR3.2*, *TaBZR4.3*, and *TaBZR6* (Figure S5).

### 2.4. Conserved Motif Analysis

We also elucidated the conserved motif of TaBZR proteins using the MEME (Multiple Em for Motif Elicitation) online servers. Finally, 10 conserved motifs were identified in 20 TaBZR genes (Figure 5A,B).

**Figure 6:**
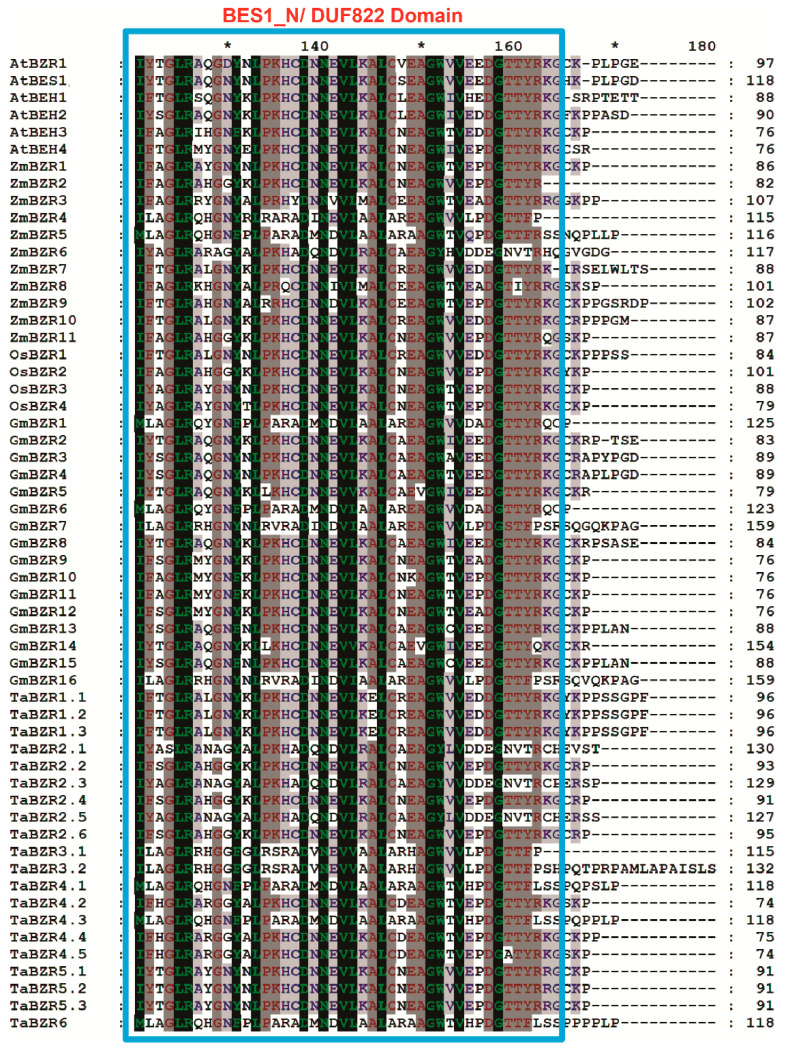
Alignment of the TaBZR with other plant species BZR protein sequences. Deduced amino sequence of TaBZR was aligned with BZR proteins from *Arabidopsis thailiana*, *Zea mays*, *Oryza sativa*, and *Glycine max*. The conserved BES_N1.DUF822 domain is boxed with light blue color. Colored and shaded amino acids are chemically similar residues. Dashes indicate gaps introduced to maximize the alignment of the homologous region. * indicates positions which have a single, fully conserved residue.

The TaBZR gene family was identified by the presence of the BES1_N domain (PF05687), and all TaBZRs had at least one BES1_N domain (Table S5), which regulates the expression of BR responsive genes and maintain the BR homeostasis in plants. The number of conserved motifs varied from 3 to 8 in the TaBZR gene family. Subfamily I had more than seven motifs, while subfamilies II, III, and IV had five motifs. These results suggest that TaBZR members clustered in the same subfamily might have a similar function. In addition, amino acid sequence alignment also revealed that all TaBZR proteins share a highly conserved BES1 domain (basic helix-loop-helix) and serine-rich phosphorylation sites at the N terminal (Figure 6 and Figure S6A). To understand the molecular function of TaBZR genes in *T. aestivum*, three-dimensional (3D) protein models of TaBZRs were generated using a phyre2 server. TaBZR 3D protein structure containing α-helical and β sheets (Figure S6B).

### 2.5. Cis-Acting Regulatory Elements (CAREs) Analysis of TaBZR Genes

**Figure 7:**
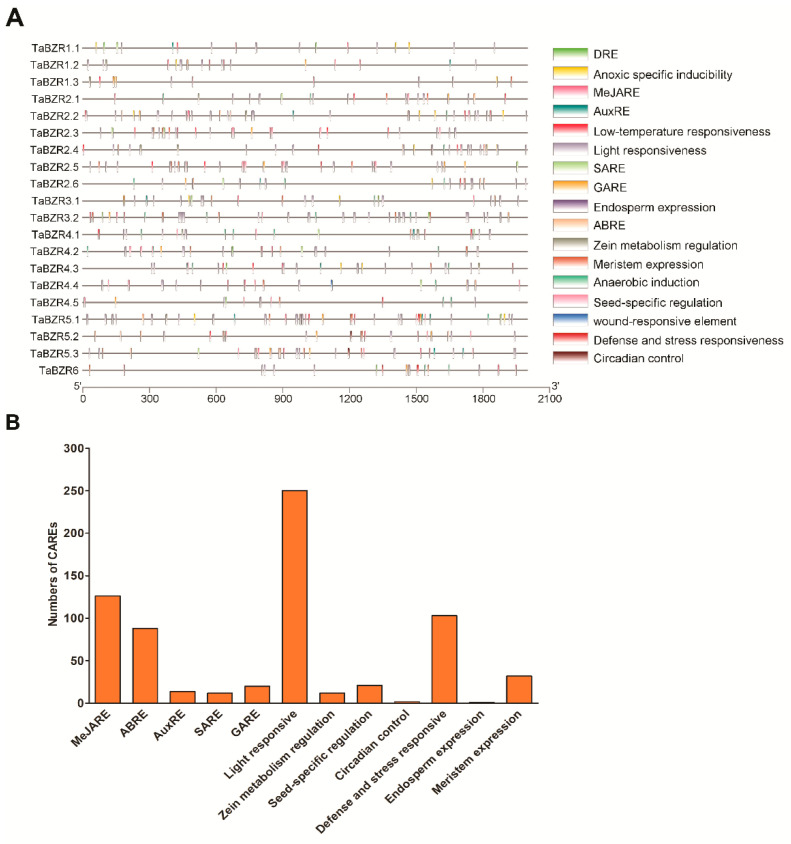
Cis-acting regulatory elements (CAREs) of the TaBZR gene family. The CAREs analysis was performed with a 2 kb upstream region using PlantCARE online server. (**A**) Hormone-responsive elements, stress-responsive elements, growth, and development-related elements, light-responsive elements, and other elements with unknown functions are shown by different colors. (**B**) Most commonly occurring CAREs in TaBZRs.

To further understand the potential regulatory mechanism of TaBZR genes, how these genes are regulated by phytohormone, various defense, and stress-responsive elements, the PlantCARE webserver was employed to find out putative cis-elements in the 2000 bp promoter region of TaBZRs. A total of 12 unique CAREs were identified in the TaBZR gene family, including elements related to light-responsive, methyl jasmonate (MeJA), abscisic acid response, auxin response, salicylic acid response, defense, and stress-responsive (Figure 7A and Table S6). CAREs involved in light responses, MeJA, abscisic acid response, auxin and defense, and stress-responsive were the most prevalent ones in the TaBZR gene family (Figure 7B).

This suggests that TaBZRs play an essential role in plant growth and developmental processes. Further, light-responsive CAREs were also abundant in the TaBZR gene family. These CAREs have been implicated in photosynthesis/non-photosynthesis-based light responses and circadian rhythm-mediated light responses. TaBZRs also had CAREs related to meristem expression and seed-specific regulation. The CAREs present in the TaBZR gene family suggests that they have been involved in diverse developmental processes regulated by hormone, light, and various developmental stages. The presence of multiple CAREs in the TaBZR genes indicates that these genes might be involved in a wide range of biological processes. Thus, these data provide valuable insights to understand the TaBZR gene family in response to different stress, phytohormone, and other developmental processes.

### 2.6. Gene Ontology (GO) Enrichment of TaBZR Genes

Gene ontology helps to understand the function of genes by examining their similarity with other species genes of known function. All TaBZRs were effectively annotated and assigned GO terms using AgriGO (Figure S7 and Table S7). TaBZRs were also annotated using eggNOG-mapper for further confirmation (Table S8), which provided similar results as AgriGO. In the biological process category, TaBZR genes enriched in the signaling (GO:0023052), response to stimulus (GO:0050896), and regulation of metabolic process (GO:0019222) categories (Figure S7A). In the cellular component category, TaBZR showed enrichment in the organelle (GO:0043226) (Figure S7B). In the molecular function category, nucleic acid-binding transcription factor activity (GO:0001071) was the most enriched category mainly involved in BR signaling (Figure S7C). In addition, the prediction of subcellular localization was carried out by BUSCO (Table 1) also showed that all the TaBZR localized in the nucleus. Apart from signaling and response to stimulus, the GO term enrichment also indicated multiple roles of TaBZR genes, including cellular response to endogenous stimulus, hormone, cell communication, chemical stimulus, and regulation of RNA biosynthetic. Therefore, these results suggest that TaBZR genes play a crucial role in plant growth and developmental processes.

### 2.7. Expression Profiling of TaBZR Genes

**Figure 8:**
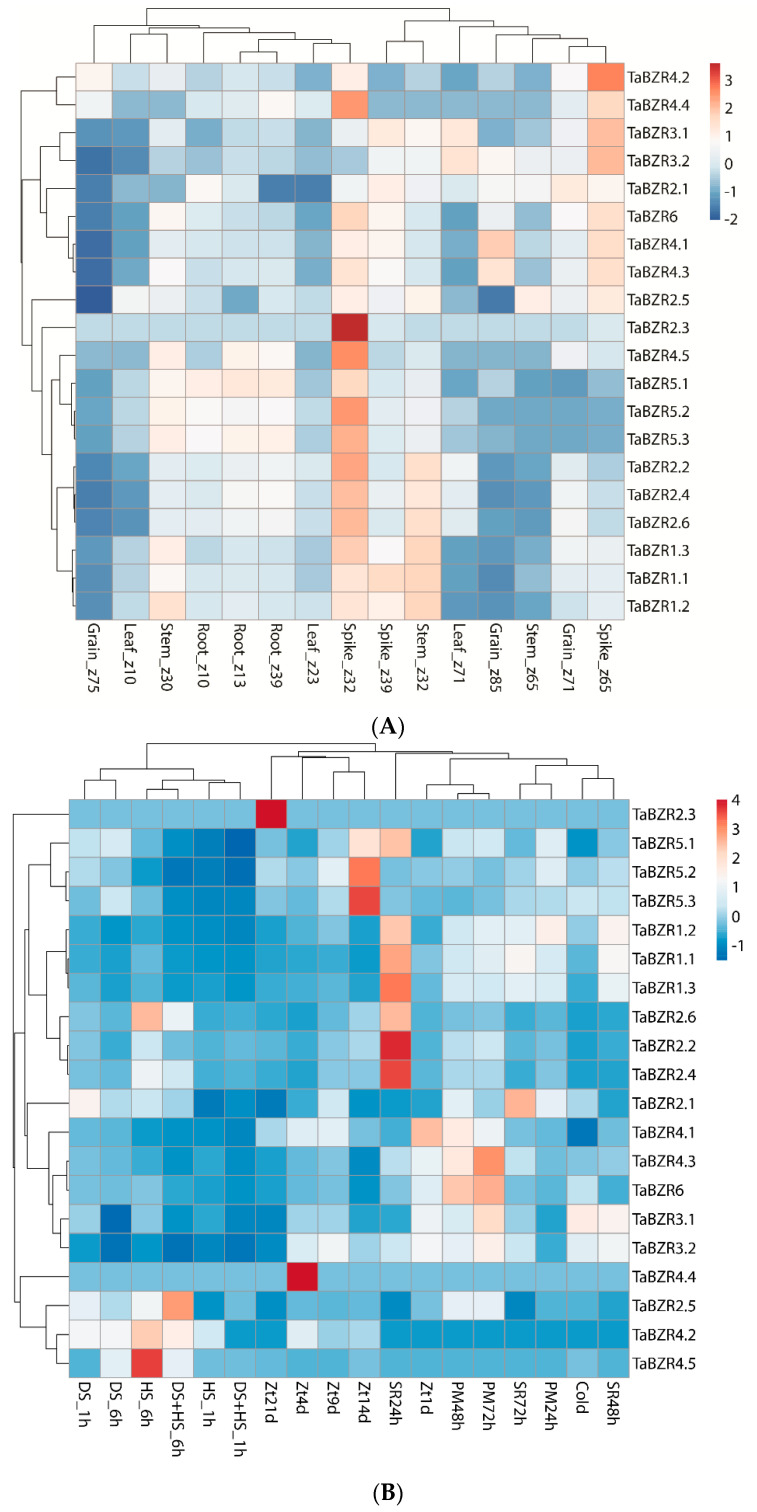
Heatmaps are representing the expression pattern of TaBZR genes. (**A**) In various developmental stages. (**B**) In different stress conditions. TPM values were directly used to construct the heatmaps. DS: drought stress, HS: heat stress, Zt: *Zymoseptoria tritici*, PM: powdery mildew; SR: stripe rust, h: hour, and d: days.

To understand the expression pattern of TaBZR genes in various development and different stress conditions, we retrieved TPM values of all TaBZRs from the wheat expression database. These TPM values were used to produce the PCA and heatmaps (Figure 8A and Figure S8). Five different tissues from the three different developmental time points were taken to determine the expression profiling of TaBZRs in this study. The time points represent in the Zadoks scale. Different TaBZR genes exhibited differential induction in the different tissues, for instance *TaBZR1.3*, *TaBZR2.2*, *TaBZR2.3*, *TaBZR2.4*, *TaBZR2.6*, *TaBZR4.4*, *TaBZR4.5*, *TaBZR5.1*, *TaBZR5.2*, *TaBZR5.3*, and *TaBZR6* displayed induction at spike z32 stage, and *TaBZR2.5*, *TaBZR3.1*, *TaBZR3.2*, *TaBZR4.1*, *TaBZR4.2*, *TaBZR4.3*, *TaBZR4.4*, and *TaBZR6* at spike z65 stage (Figure 8A).

The expression of *TaBZR1.1*, *TaBZR1.2*, *TaBZR1.3*, *TaBZR2.2*, *TaBZR2.4*, and *TaBZR2.6* were elevated in the stem at the z32 stage, while *TaBZR4.1* and *TaBZR4.3* were also up-regulated in grain at the z85 stage, respectively. *TaBZR5.1* and *TaBZR5.3* showed induction in root at z13 and z39 stages, respectively. In addition, *TaBZR3.1* and *TaBZR3.2* expressions were raised in leaf at the z71 stage. Thus, these results demonstrated that different TaBZRs might participate in developing different tissues at different stages.

**Figure 9:**
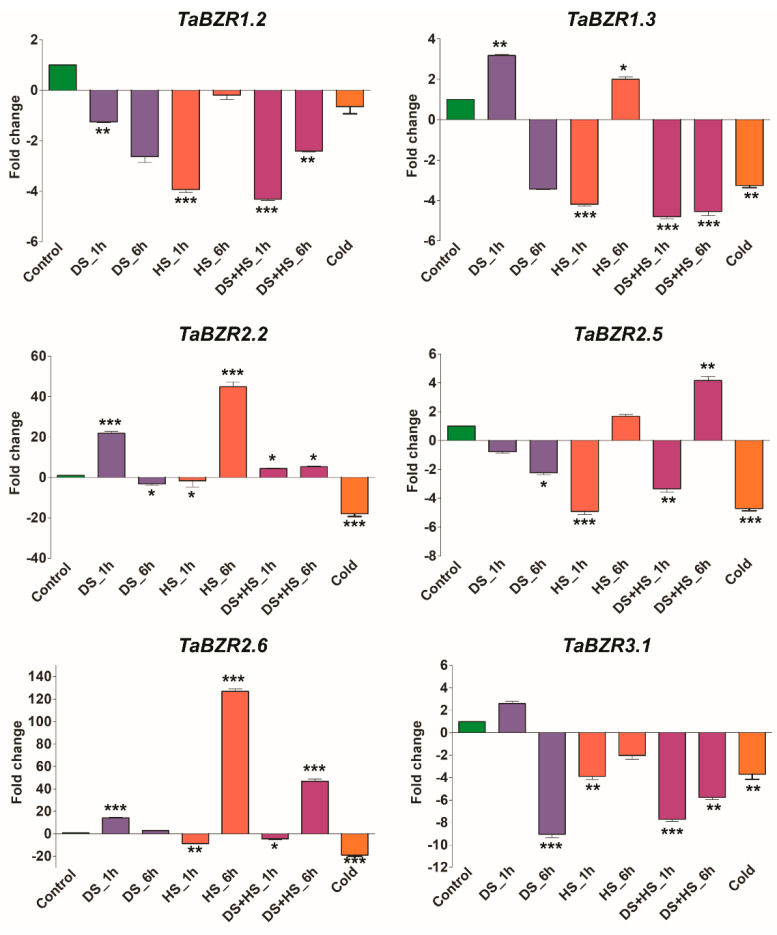

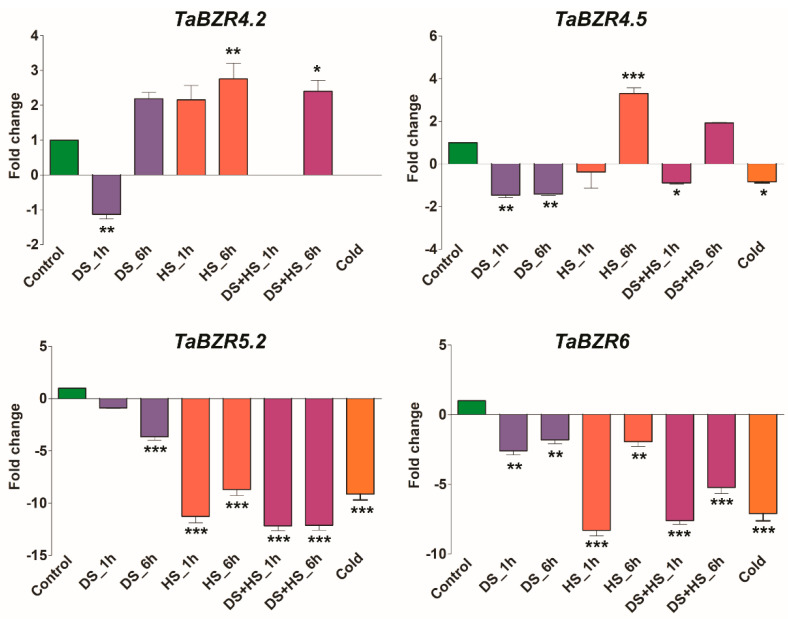
Quantitative real-time PCR analysis of selected TaBZR genes in response to drought, heat, and cold stress to verify RNA-seq data. The wheat actin gene was used as Table S9 and <0.001 level (* *p <* 0.05, ** *p* lies in between the values of 0.05 and 0.001, and *** *p <* 0.001). Error bars show standard deviation. Data are mean ± SD (*n* = 3).

Expression profiling of TaBZRs was also examined under six different stress conditions as biotic (septoria tritici blotch, stripe rust, and powdery mildew) and abiotic stress (drought, heat stress, and cold). Several members of the TaBZR family were observed to be induced during biotic and abiotic stress (Figure 8B). *TaBZR1.1*, *TaBZR1.2*, *TaBZR1.3*, *TaBZR2.2*, *TaBZR2.4*, *TaBZR2.6*, and *TaBZR5.1* were highly induced upon stripe rust infection (pathogen CRY31). *TaBZR5.1*, *TaBZR5.2*, and *TaBZR5.3* were significantly elevated after the 14 days of infection of *Zymoseptoria tritici*, while the expression of *TaBZR4.4* and *TaBZR2.3* were highly up-regulated after the 4 and 21 days infection of *Zymoseptoria tritici*, respectively. *TaBZR3.1*, *TaBZR4.3*, and *TaBZR6* were showed higher induction after the 72 h infection of powdery mildew (pathogen E09). In the case of abiotic stress, data indicate that the expression of some members of the TaBZR family, such as *TaBZR2.6*, *TaBZR4.2*, and *TaBZR4.5*, were induced during the heat stress while *TaBZR3.1* in cold stress. It seems that the TaBZR family is not involved in drought stress. However, *TaBZR2.5* was up-regulated during the combined heat and drought stress (Figure 8B). However, the majority of TABZR genes were down-regulated in the biotic and abiotic stresses. Expression patterns of some selected TaBZR genes were also verified by qRT-PCR. qRT-PCR results showed almost similar expression trends with slight variation (Figure 9). Overall, the expression pattern was consistent across the two different approaches. Collectively, these results demonstrated that TaBZR family members were involved in response to diverse stress.

### 2.8. Protein-Protein Networks Analysis of the TaBZR Family Genes

**Figure 10:**
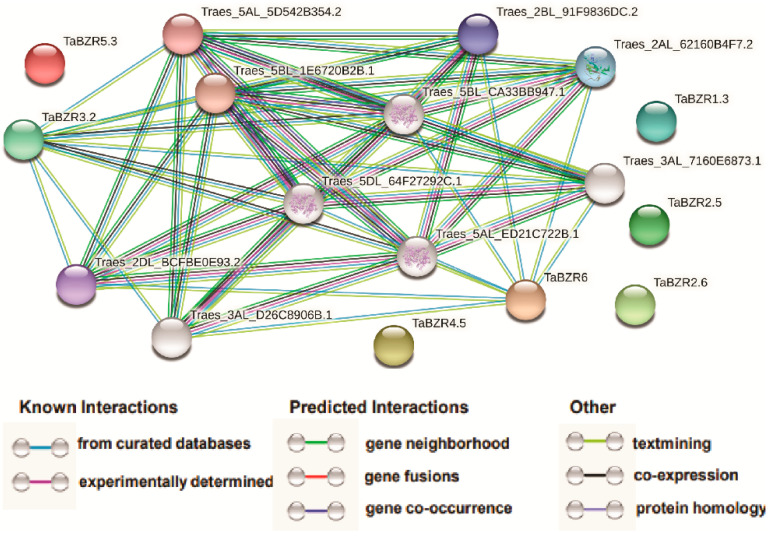
Protein-protein interaction analysis of TaBZRs proteins. Protein-protein interaction network produced by STRINGV9.1. Each node represents a protein, and each edge represents an interaction, colored by evidence type.

To investigate protein-protein interactions between TaBZRs and other wheat proteins, a network was constructed using the STRING database (Figure 10 and Table S9). According to the predicted results, we identified two TaBZRs interacting with 10 different *T. aestivum* proteins. TaBZR3.2 can interact with 10 other *T. aestivum* proteins such as Traes_2AL_62160B4F7.2, Traes_2BL_91F9836DC.2, Traes_2DL_BCFBE0E93.2, Traes_3AL_7160E6873.1, Traes_3AL_D26C8906B.1, Traes_5BL_CA33BB947.1, Traes_5DL_64F27292C.1, Traes_5AL_ED21C722B.1, Traes_5AL_5D542B354.2, and Traes_5BL_1E6720B2B.1, while TaBZR6 can interact with eight other *T. aestivum* proteins Traes_2AL_62160B4F7.2, Traes_2BL_91F9836DC.2, Traes_2DL_BCFBE0E93.2, Traes_3AL_7160E6873.1, Traes_3AL_D26C8906B.1, Traes_5AL_ED21C722B.1, Traes_5BL_CA33BB947.1, and Traes_5DL_64F27292C.1, which were α-Amylases 2-related and starch phosphorylase. α-Amylases catalyze the hydrolysis of the α-1,4-glycosidic bonds in starch, resulting in convert starch into glucose, maltose, and maltotriose [58,59]. Starch phosphorylase is an enzyme that catalyzes the reversible transfer of glucosyl from glucose-1-phosphate to the α-1,4-D-glucan chains [60,61]. These results provided a piece of valuable information for the further functional characterization of TaBZR genes.

## 3. Discussion

BRs are plant-specific steroidal that regulates the expression of BR responsive genes in plants [13,14,21]. BZR family proteins involved in multiple developmental processes including cell division and elongation, vascular differentiation, seed germination, flowering, stomata formation, senescence, photomorphogenesis and environmental stimuli [2,11,15,21,38,41,42,44,47,62]. BZR gene family members have been reported in several plant species [31,32,33,34,35,36]. To understand how BRs regulate plant developmental processes and under different biotic and abiotic stress conditions, to this aim, the genome-wide identification of the TaBZR gene family would be necessary to dissect the intricate BR transcriptional networks. In the present study, a systematic in silico analysis was carried out for the identification and characterization of the BZR gene family in the wheat genome. A total of 20 TaBZR genes were identified in the wheat genome (Table 1). The BES1/BZR gene family has been extensively studied in *A. thaliana*, *O. sativa*, *Z. mays*, *S. lycopersicum*, *G. hirsutum*, and *B. rapa* [22,32,34,35,43]. In Arabidopsis, tomato, and Chinese cabbage, the BZR gene family is classified into three subfamilies. In our study, phylogenetic analysis revealed that the TaBZR gene family is divided into five subgroups or subfamilies (I-V). Group III and IV included the maximum members (8 and 9) of TaBZR genes, whereas none of the members were found in groups I and II. The results indicated the multiple duplications of BZR genes in different plant species. Interestingly, group V consists of monocot-specific TaBZRs (Figure 1). The origin of this type of gene possibly has the monocot-specific functions that might have an impact on physiological and morphological establishment such as phyllotaxy or development of the distinctive root system [63]. Although, TaBZR genes were well allocated into the known group of Arabidopsis, maize, rice, and soybean BZRs, suggesting that TaBZRs might originate from a common ancestor. Further, the majority of the TaBZRs exhibited orthologous relationships with Arabidopsis, maize, rice, and soybean. The phylogenetic tree also showed that groups III and IV had an expanded number of members (Figure 1), indicating that duplications occurred of these TaBZRS during the evolution. The overall evolution pattern showed lineage-specific expansion of the TaBZR gene family through the partial modification of the genome that might reinforce the adaptation to internal and external stimuli [64,65].

The wheat BZR gene family is extensively expanded and had relatively more BZRs compared to the previously reported BZRs in *A. thaliana*, *Z. mays*, *G. max*, *C. cajan*, *M. sativa*, *C. arietinum*, *S. lycopersicum*, and *B. vulgaris* [31,32,33,34,36,66,67]. The gene duplication processes, including segmental, tandem, and whole-genome duplications, are the main driving forces of the expansion of the gene family in different plant species [67,68]. Thus, these results indicating that the *BES1/BZR* gene family may play a crucial role in the evolutionary process of plants. Chromosomal mapping of TaBZR genes revealed that the 20 TaBZRs were not uniformly distributed on 21 chromosomes (Figure 2A,C). The gene number on each chromosome varied from one to two, chromosomes 2A, 2B, 2D, 4B, 4D, 6D, 7A, 7B, and 7D having a single gene, while chromosome 3A, 3B, 3D, 6A, and 6B had two genes. Gene duplication analysis showed the seven pairs of duplicated genes, which shared high nucleotide sequence similarity. The duplicated pairs are *TaBZR1.1*:*TaBZR1.2, TaBZR2.1*:*TaBZR2.3*, *TaBZR2.2/TaBZR2.6*, *TaBZR3.1*:*TaBZR3.2*, *TaBZR4.2*:*TaBZR4.4*, *TaBZR4.3*:*TaBZR6*, and *TaBZR5.1*:*TaBZR5.2*. Further, the Ka/Ks ratios of seven gene pairs were less than 1, revealing that TaBZR genes underwent a robust purifying selection pressure (Figure S4 and Table S3). The expansion of the TaBZR gene family might be due to the natural whole-genome duplication events. Our gene duplication analysis revealed that the TaBZR gene duplication events were similar as previously described in the maize, *B. rapa*, soybean, chickpea, and Medicago [31,43,66]. Therefore, these results demonstrated that whole-genome duplication and segmental duplications might play an important role in the expansion and evolution of the TaBZR genes. To examine the synteny relationships of BZR genes in the wheat and different plant species, we identified 22, 17, 42, 20, and 26 orthologous gene pairs between TaBZRs with other BZR genes in *B. distachyon*, *Ae. tauschii*, *T. dicoccoides*, *O. sativa*, and *A. thaliana*, respectively (Figure 3 and Table S4). In addition, *B. distachyon* (BB, diploid) and *Ae. tauschii* (DD, diploid) were the natural foundation of B and D subgenomes of hexaploid wheat. The synteny relationship revealed that five orthologous gene pairs between *Ae. tauschii* with a wheat D subgenome were located on the same chromosomes, one on 2D, two on 3D, one on 4D, and one on 7D (Figure 3 and Table S4). Further, 12 orthologous gene pairs between *T. dicoccoides* with a wheat AABB subgenome were located on the same chromosomes with one on 2A, two on 3A, two on 6A, one on 7A, two on 3B, one on 4B, two on 6B and one on 7B (Figure 3 and Table S4). These results indicate that these BZR genes might be derived from *Ae. tauschii* and *T. dicoccoides* orthologous genes during hybridization events. Moreover, more orthologous gene pairs were identified between wheat with *O. sativa* and *A. thaliana*, which showed that TaBZR and other BZR genes in these plant species might be originated from a common ancestor during the evolutionary process.

The gene structure analysis of TaBZRs demonstrated that gene structure is highly conserved for most of the TaBZR genes. A total of 15 out of 20 TaBZR genes contain 2 exons (Figure 4). *TaBZR3.2*, *TaBZR4.3*, and *TaBZR6* had nine exons, while *TaBZR3.1* and *TaBZR4.1* contain eight and seven exons, respectively. Intron size is a key factor that influences the gene size; for instance, the remarkable difference in gene size was observed between the biggest gene *TaBZR6* (4.8 kb) and the smallest gene *TaBZR2.1* (0.1 kb) was due to the difference in total intron length (4.8 vs. 0.1 kb). This conserved exon/intron organization of BZR genes was also found in other plant species [31,32,33,36]. We identified the 10 conversed motifs to elucidate structural comparisons among TaBZR proteins (Figure 5A,B). Conserved motif analysis also revealed that two distinct types of motif compositions were observed among the TaBZR proteins. We found that four motifs were common in all the TaBZR proteins (Motif 1, 3, 5, and 7). The genes of the same subgroup commonly have the same motifs and are more conservative. Motif 1, 3, and 10 consists of DUF822 domain (PF05687), classified as BES1/BZR1, a plant-specific transcription factor that coordinates with transcription factors, for instance, BIM1 to regulate brassinosteroid responsive genes [11,12,13,14], while motif 2, 6, 7, 8, and 9 belong to the glycosyl hydrolase family 14 (PF01373). Glycoside hydrolases are the most common group of enzymes that cleave the glycosidic bond between more than two carbohydrates and a non-carbohydrate moiety [34,69]. Further, motif 5 possesses the DUF3660 domain (receptor serine/threonine kinase); this domain is mainly found in eukaryotes with conserved ELPL sequence motif, annotated with the domain of an unknown function. Therefore, we assume that genes of the same subgroup might have a similar function. In addition, multiple sequence alignment of TaBZR with other plant species BZR proteins also displayed highly conserved basic helix-loop-helix (bHLH) or BES1 domain (DUF822), glycosyl hydrolase14 domain, and serine-rich C-terminal (Figure 6 and Figure S6). TaBZR family contains both the DUF822 and glycosyl hydrolase 14 domains such as *TaBZR3.1*, *TaBZR3.2*, *TaBZR4.1*, *TaBZR4.3*, and *TaBZR6*, while *TaBZR1.1*, *TaBZR1.2*, *TaBZR1.3*, *TaBZR2.1*, *TaBZR2.2*, *TaBZR2.3*, *TaBZR2.4*, *TaBZR2.5*, *TaBZR2.6*, *TaBZR4.2*, *TaBZR4.4*, *TaBZR4.5*, *TaBZR5.1*, *TaBZR5.2*, and *TaBZR5.3* contain only DUF822 domain. A previous study in the Chinese cabbage and tomato showed two conserved domains, including DUF822 and glycosyl hydrolase14, among DUF822, the highly conserved domain (110–148 aa) located at the N terminal of the protein [34,69]. The BES1/BZR family in Arabidopsis, rice, and cotton only had the DUF822 domain [34,66]. These results indicate that TaBZR may have a conserved and redundant function in wheat. In Arabidopsis, a single mutant of AtBZR1, AtBES1, and four homologs AtBEH1–4 did not exhibit any phenotype. However, tapetum and microsporocyte development defects in anthers were observed in the quintuple mutant (bes1/bzr1/beh1/beh3/beh4) and sextuple mutant (bes1/bzr1/beh1/beh2/beh3/beh4), suggesting the highly functional redundancy of BZR genes [37,38]. Therefore, we could speculate that multiple mutants of TaBZR genes may also affect the same cellular processes in wheat. The determination of the subcellular localization of TaBZR proteins will help understand the molecular function. All TaBZR proteins were predicted to locate in the nucleus (Table 1). The nucleus localized BZRs have been regulating the expression of BR responsive genes [28,29,30]. Further, 3D protein structure analysis also showed that wheat BZR proteins consist of α-helix and curl structure, which is mainly distributed in the nucleus; we assume that BZR genes play a critical role in signal transduction to regulate the downstream signaling pathways in the wheat. Collectively, these results are suggesting that BZR genes in wheat may have a similar function.

The cis-acting regulatory element is a non-coding DNA sequence that exists in the promoter regions of a gene. The distribution of other CAREs in promoter regions may reveal differences in the regulation and function of genes [70]. The identified CAREs elements in this study were classified into three main categories: phytohormone response, stress response, growth, and development (Figure 7). More than five CAREs were identified in the promoter region of each TaBZR (Table S6). A total of 16 cis-elements related to light response were detected, such as Box 4 and AE-box (part of a conserved DNA module involved in light-responsiveness), Box II, TCT-motif, GTGGC-motif, I-box, chs-CMA1a, chs-CMA2a, L-box, GATA-motif, and TCCC-motif (part of a light-responsive element), ACE and G-box (cis-acting element involved in light-responsiveness) and Sp1, MRE and GT1-motif (light-responsive element) [71,72]. We also predicted five cis-elements related to growth and development including CAT-box (meristem expression), RY-element (seed-specific regulation), CAAAGATATC-motif (circadian control), GCN4-motif (endosperm expression), and O2-site (zein metabolism regulation) [73,74]. Further, we also examined the CARE related to hormone response in the promoter of TaBZR genes. The ABRE cis-elements were predicted in all TaBZR genes except TaBZR1.1 and TaBZR4.3. Subsequently, we also found some other hormone-related cis-elements such as TCA-element (salicylic acid responsiveness), P-box and TATC-box (gibberellin-responsive element), CGTCA-motif (MeJA-responsiveness), TGA-element and AuxRR-core (auxin-responsive element) [75]. Moreover, other cis-elements that have been implicated in diverse stress conditions, including MBS (drought inducibility), LTR (low-temperature responsiveness), and TC-rich repeats (defense and stress responsiveness), were also predicted in the TaBZR promoters. Gibberellin plays an important role in cell division, seed germination, stem elongation, flower development, leaf, and fruit senescence [2,27,76]. The previous studies have demonstrated that the promoters of GA2ox-3, GA3ox-2, GA20ox-2, and D2 contain the G-box, BRRE, and CATGTG elements; BZR genes regulate the expression of downstream genes, thereby influencing GA synthesis [2,76]. Further, previous studies have shown that differential expression of BZR gene family members under different hormone treatment in tomato, sugarbeet, and apple [33,36,77,78]. Similarly, the expression of SlBES1.8 was induced by auxin and gibberellin [33], while the expression of GhBES1, BoBES1, and MdBES1 genes was raised after BR treatment [22,78]. Remarkably, all TaBZR contain phytohormone, growth, and stress-responsive CAREs in their promoter sequences. Taken together, these results demonstrated that BZR gene family members in wheat might be regulated by a wide range of developmental processes, various hormones, and stress; of course, this needs to be confirmed in the future by experimental studies. These data will provide valuable insights to understand the TaBZR gene family in response to plant developmental processes, phytohormone, and different biotic and abiotic stresses. Furthermore, the application of genome editing technology might lead to a better understanding of the function of TaBZR genes. Therefore, we hypothesize that TaBZR genes are involved in the plant growth and development of wheat and significantly improve plant tolerance to diverse biotic and abiotic stresses.

Several studies have been demonstrated the role of BZR proteins in numerous developmental processes and response to environmental stimuli [11,13,14,15,18,22,25,29,39,41,44,45]. Expression profiling of the TaBZR gene family revealed that TaBZR expression was observed in different tissues such as *TaBZR1.3*, *TaBZR2.2*, *TaBZR2.3*, *TaBZR2.4*, *TaBZR2.5*, *TaBZR2.6*, *TaBZR3.1*, *TaBZR3.2*, *TaBZR4.1*, *TaBZR4.2*, *TaBZR4.3*, *TaBZR4.4*, *TaBZR4.5*, *TaBZR5.1*, *TaBZR5.2*, *TaBZR5.3*, and *TaBZR6* were highly expressed in the spike (Figure 8A). *TaBZR1.1*, *TaBZR1.2*, *TaBZR1.3*, *TaBZR2.2*, *TaBZR2.4*, and *TaBZR2.6* were up-regulated in the stem. Further, *TaBZR3.1* and *TaBZR3.2* showed induction in the leaf. In addition, *TaBZR4.1* and *TaBZR4.3* expressions were raised in the grain, while *TaBZR5.1* and *TaBZR5.3* showed higher expression in the root. The PCA plot of various developmental stage expression patterns also indicates that TaBZRs are involved in leaf, stem and root, grain development. Subsequently, these tissue expression profiles cluster together (Figure 8A and Figure S8A). Many previous studies showed the function of BZR in a variety of developmental processes, including cell division and elongation, vascular differentiation, seed germination, flowering, stomata formation, senescence, and photomorphogenesis [11,13,14,15,25,29,39,41,44,45]. Similarly, MdBES1 genes level were highly elevated in leaves, flowers, young and mature fruits [78]. Further, the *SlBES1.8* gene is highly expressed in floral organs in tomatoes [33]. Overexpression of the *AtDWF4* in *Brassica napus* exhibited increased root length, higher root biomass, seed yield, and tolerance to biotic and abiotic stress [39]. BRI1 gene independently regulates anther development in Arabidopsis. In addition, BR also regulates hypocotyl cell expansion and promotes the transition from meristematic cells to primordial cells in the shoot [28,62,76,79]. In contrast, in the root apex, BRs regulates root growth by coordinating root cell elongation and meristem size [18,80,81,82]. Furthermore, overexpression of *GmBZL2* enhanced BR signaling, resulting in increased seed number per silique, while mutant lines exhibited curly leaves and delayed flowering in Arabidopsis [42]. In all of these cellular processes, the BZR gene family plays an important role by regulating numerous transcriptional factors to integrate other signaling pathways. Moreover, our gene ontology analysis also suggested multiple roles of the TaBZR gene in the cell. Interestingly, in our investigation, TaBZR gene family members are also expressed in various tissue and developmental stage in wheat. Therefore, this temporal and spatial expression pattern of TaBZR genes indicates that these BZRs may play a crucial role in development of various tissues and organ in wheat.

BZR gene family plays a critical role in plants’ adaptations to internal and external stimuli at both transcriptional and post-transcriptional levels [17,18,19,20,21]. Our results also showed that seven TaBZRs genes (*TaBZR1.1*, *TaBZR1.2*, *TaBZR1.3*, *TaBZR2.2*, *TaBZR2.4*, *TaBZR2.6*, and *TaBZR5.1*) were highly induced upon stripe rust infection (Figure S9), while the expression of five TaBZR genes (*TaBZR2.3*, *TaBZR4.4*, *TaBZR5.1*, *TaBZR5.2*, and *TaBZR5.3*) were significantly elevated during *Zymoseptoria tritici* infection. Further, *TaBZR3.1*, *TaBZR4.3*, and *TaBZR6* were showed higher induction after the 72 h infection of powdery mildew. *TaBZR2.6*, *TaBZR4.2*, and *TaBZR4.5* were induced during the heat stress, while *TaBZR3.1* was in cold stress. *TaBZR2.5* was responded to the combined heat and drought stress (Figure S9). The PCA plot of different stress expression patterns also indicates that TaBZRs are involved in these stress; hence, drought and heat expression profiles cluster detached from other expression profiles (Figure 8B and Figure S8B). However, most of the TaBZR genes are down-regulated in the responses to biotic and abiotic stresses. These results indicate that TaBZR genes may negatively regulate genes involved in response to diverse biotic and environmental stresses. Hence, we assume that knockdown of TaBZR gene family members may enhance the stress tolerance and thus improve the quality and increase wheat yield. Previous studies have shown that BZR genes have distinct and different responses to biotic and abiotic stresses [22,32]. AtBZR1 suppresses the expression of hydrogen peroxide-induced NAC transcription factor (JUNG BRUNNEN1, JUB1) that functioned in different abiotic stress tolerance [69,83]. Likewise, the BR signaling pathway also hinders drought response by negatively regulating BES1 and RD26 (RESPONSIVE TO DESICCATION 26), resulting in the repression of the genes involved in the BR pathway [41]. SlBES1 genes were partly induced and repressed after PEG treatment in tomatoes [32]. Additionally, the expression of BZR genes was repressed when tomato seedlings were exposed to diverse stresses, including salt, drought, dehydration, osmosis, and wound stress [33]; hence, they might negatively regulate genes involved in biotic and abiotic stresses. All BrBZRs exhibited highly elevated against ABA treatment; however, they showed differential expression under cold, drought, and salt stress [43]. Similarly, differential expression patterns of BZR genes were observed under salt, cold BR, SA, and MeJA stress in Eucalyptus [77]. Six ZmBZRs displayed higher expression under hypoxia, N starvation, and salt stress, but their expression levels were reduced in high-temperature stress [31]. Similar results were also observed for BZR genes in other plant species. Moreover, expression profiling of TaBZRs was further validated by qRT-PCR. qRT-PCR results also exhibited a similar expression with slight variation (Figure 9). The expression pattern of TaBZR genes under biotic and abiotic stress revealing that they might involve in the stress tolerance in wheat. Thus, these results demonstrated that wheat responds to diverse stresses through an intricate gene network, which requires coordinated regulation among the TaBZRs.

Many studies have been demonstrated that TaBZR interacts with other proteins to regulate diverse developmental processes, hormonal and stress responses. BZR1/BES1 transcription factors bind to the BR response element (CGTGT/CG) or E-box (CANNTG) to regulate the expression of thousand target genes [28,29,30]. Our STRING database predicted results also revealed that TaBZR3.2 and TaBZR6 interact with different *T. aestivum* proteins (Figure 10 and Table S9); however, TaBZR3.2 and TaBZR6 were co-expressed with TaBZR2.5, TaBZR3.1, TaBZR4.1, TaBZR4.2, TaBZR4.3, and TaBZR4.4 in spike and leaf (Figure 8A), suggesting that TaBZR3.2 might perform essential functions in spike and leaf development through interacting with each other. TaBZR genes also interacted with the α-Amylases 2-related and starch phosphorylase proteins. α-Amylases (AMY) and starch phosphorylase play an important role in starch metabolism [58,59,60,61]. α-Amylases 3 is strongly associated with BRI1, which controls the starch degradation in Arabidopsis [84]. BZR1-BAMs (β-Amylase-like proteins: BAM7 and BAM8) also regulates plant growth and development via a crosstalk with BR signaling [85,86]. Our findings also indicate that TaBZR might function in plant growth and development and diverse stress tolerance by participating in BR signaling processes. In summary, our work provides valuable information about the TaBZR gene family, their functions in the various plant developmental processes, and response to hormone and multiple stresses. Therefore, the results of this study are significant to elucidate further the precise role of the TaBZR gene family in plant growth and development and diverse stress conditions.

## 4. Materials and Methods

### 4.1. Identification of BZR Genes in the Wheat Genome

To perform genome-wide analysis in bread wheat, genome data (IWGSC) were retrieved from the Ensembl Plants website (http://plants.ensembl.org/index.html accessed on 10 May 2021). To identify putative BZR genes in wheat, we created a local database of the protein sequences of bread wheat in BioEdit ver. 7.2.6 [87]. The thirty-seven BZR genes from Arabidopsis, rice, maize, and soybean were used as queries to identify putative BZR genes in bread wheat in a local database using BLASTp. The e-value of 10−5 and > 100-bit scores were kept cut off to identify putative BZR genes, and finally, the BLASTp output was tabulated. Based on the above method, putative BZR candidates were selected. After removing redundant results, the remaining sequences were further verified for the existence of BES1_N domains using other databases: Simple Modular 132 Architecture Research Tool tool (SMART, http://smart.emblheidelberg.de/ accessed on 10 May 2021), InterPro (https://www.ebi.ac.uk/interpro accessed on 10 May 2021) NCBI CDD (https://www.ncbi.nlm.nih.gov/Structure/cdd/cdd.shtml accessed on 10 May 2021) and HMMscan (https://www.ebi.ac.uk/Tools/hmmer/search/hmmscan accessed on 10 May 2021) and the sequences lacking BES1_N domains were removed. Finally, the protein sequences with BES1_N domains were taken and named sequentially according to their locations on the chromosomes.

### 4.2. Chromosome Localization and Gene Duplication

For the distribution of chromosomes, genomic positions of BZR genes were downloaded from Ensembl Plants biomart (http://plants.ensembl.org/biomart/martview accessed on 11 May 2021). The BZR genes were named with a ‘Ta’ prefix and numbered in ascending order with their increasing position on the chromosome. PhenoGram (http://visualization.ritchielab.org/phenograms/plot accessed on 11 May 2021) was used to represent TaBZR genes on the wheat chromosomes. MCScanX tool kit was used to investigate gene duplication events within species and the similarity between BZR genes in wheat and other plant species [88]. The non-synonymous substitution rate (Ka), synonymous substitution rate (Ks), and the Ka/Ks ratio were calculated using the TB tools [89].

### 4.3. Physico-Chemical Characteristics, Subcellular Localization, and 3D Structure

The protein characteristics, including isoelectric point, lengths, and molecular weight of TaBZR proteins, were evaluated by isoelectric point calculator [90] and ExPASy (https://web.expasy.org/compute_pi/ accessed on 15 May 2021). Subcellular localization was predicted using CELLO (http://cello.life.nctu.edu.tw/ accessed on 15 May 2021) and BUSCA (http://busca.biocomp.unibo.it/ accessed on 15 May 2021). The three-dimensional (3D) structure of TaBZRs was produced using the Phyre2 server (http://www.sbg.bio.ic.ac.uk/phyre2/html/page.cgi?id=index accessed on 15 May 2021).

### 4.4. Gene Structure, Gene Ontology, and Motif Analysis

The CDS, genomic, and protein sequences of wheat BZR genes (Table S1) were retrieved from the Ensembl Plants biomart (http://plants.ensembl.org/biomart/martview accessed on 18 May 2021). Intron, exon positions, and untranslated region were visualized using the Gene Structure Display Server 2.0 (http://gsds.gao-lab.org/ accessed on 18 May 2021). For gene ontology, TaBZR protein sequences were used to predict gene ontology terms using agriGO [91] and EggNOG (http://eggnogdb.embl.de/#/app/emapper accessed on 19 May 2021). The conserved motifs in the TaBZR were elucidated using the MEME tool (http://meme-suite.org/tools/meme accessed on 19 May 2021) with default settings.

### 4.5. Cis-Acting Regulatory Elements (CAREs) Analysis and Protein Interaction Network

For identification of CAREs, 2000 bp upstream sequences of BZR genes (Table S1) were downloaded from Ensembl Plants and analyzed using PlantCARE online server (http://bioinformatics.psb.ugent.be/webtools/plantcare/html/ accessed on 20 May 2021). The number of occurrences for each CARE motif was counted for TaBZR genes, and the most commonly occurring CAREs were used to make Figure 7A in TBtools [89]. The TaBZR protein interaction network was examined using the STRING online server (https://string-db.org/cgi accessed on 20 May 2021).

### 4.6. Expression Profiling of TaBZR Genes

Transcripts per million (TPM) value for five tissues (leaf, stem, root, spike, and grain) and under diverse stress conditions were retrieved from Wheat Expression Browser (http://www.wheat-expression.com/ accessed on 21 May 2021). Heatmaps and principal component analysis (PCA) plots were produced using ClustVis (https://biit.cs.ut.ee/clustvis/ accessed on 21 May 2021) and TBtools software [89].

### 4.7. Plant Material, Growth Conditions, Drought, and Heat Treatment

Wheat (*Triticum aestivum* L.) cv. HI 1612, an Indian cultivar suitable for restricted irrigated conditions, tolerant to heat stress, resistant to stripe, leaf rusts, leaf blight, and loose smut, was used for the experiments. Seeds of HI 1612 were sown on soil in plastic pots and reared in a greenhouse. Ten-day-old wheat seedlings were acclimatized for two days in growth chamber conditions. They were further subjected to drought and high-temperature stress (37 °C) for 1 and 6 h, while cold stress for 3 days (4 °C) [92]. Controls were kept at 25 °C. The cold, drought, and high-temperature stressed seedlings were collected for RNA extraction and stored at −80 °C.

### 4.8. RNA Isolation and Real-Time PCR

RNA was isolated from control, cold, drought, and heat-treated plants as described by [93,94]. To remove the genomic DNA contamination from RNA samples were treated with DNase I (Takara Bio. Inc., Shiga, Japan) at 37 °C for 30 min. cDNA was prepared using the iScriptTM cDNA synthesis kit (Bio-Rad, Hercules, CA, USA) at 46 °C for 20 min. cDNA was quantified using a NanoDrop (ND-1000, NanoDrop Technologies, Wilmington, DE, United States). Quantitative real-time PCR (qRT-PCR) was performed using the Applied Biosystems 7500 Fast Real-Time PCR (Applied Biosystems, Massachusetts, United States). Each qRT-PCR reaction was carried out with three technical replicates and repeated three times. The qPCR reactions were run with the following profile: denaturation at 95 °C for 1 min, followed by 39 cycles of denaturation at 95 °C for 15 s, and annealing at 55–60 °C for 30 s. A melting curve was obtained through a protocol of 95 °C for 1 min, 95 °C for 15 s, 65 °C for 1 min followed by cooling at 40 °C for 10 min. The fold change was calculated based on mean 2^−ΔΔCT^ values [95,96,97]; finally, this value was used for plotting graphs. Wheat actin (AB181991) was used as the internal control to normalize the data. Primer pairs were designed using PrimerQuest Tool (https://sg.idtdna.com/PrimerQuest/Home/Index accessed on 17 May 2021), and primers used in this work are listed in Table S10.

## 5. Conclusions

Bread wheat is an important cereal crop and staple food worldwide. Thus, all plant scientists have been concentrated on improving the quality, increasing yield, and stress tolerance in wheat. BZR gene family has been involved in plant growth and development. In the present study, we identified and characterized the BZR gene family in wheat. Expression profiling also showed the role of TaBZRs in various developmental stages and stress conditions. Therefore, this study provides putative candidate genes for improving plant growth, stress tolerance and also facilitates a better understanding of the various developmental processes in bread wheat.

## Figures and Tables

**Table 1:** Nomenclature and characteristics of the putative brassinazole-resistant (BZR) proteins in wheat.

Proposed Gene Name	Gene ID	Genomic Location	Orientation	CDS Length (bp)	Intron Number	Protein Length (aa)	Molecular Weight (KDa)	pI	GRAVY	Predicted Subcellular Localization
*TaBZR1.1*	*TraesCS2A02G187800*	2A:150124286-150124643	Reverse	942	1	313	33.68	7.7	−0.5881	Nucleus
*TaBZR1.2*	*TraesCS2B02G219300*	2B:209436846-209437190	Forward	942	1	313	33.61	7.7	−0.583	Nucleus
*TaBZR1.3*	*TraesCS2D02G199900*	2D:151325825-151326183	Forward	942	1	313	33.62	7.7	−0.5859	Nucleus
*TaBZR2.1*	*TraesCS3A02G123500*	3A:99588221-99588358	Forward	558	1	185	19.96	8.8	−0.4816	Nucleus
*TaBZR2.2*	*TraesCS3A02G139000*	3A:116518978-116520359	Forward	1071	1	356	37.41	8	−0.5098	Nucleus
*TaBZR2.3*	*TraesCS3B02G142600*	3B:130257621-130257758	Forward	549	1	182	19.75	8.8	−0.6978	Nucleus
*TaBZR2.4*	*TraesCS3B02G156600*	3B:149453779-149455098	Forward	1065	1	354	37.28	8	−0.5166	Nucleus
*TaBZR2.5*	*TraesCS3D02G125100*	3D:83703425-83703710	Forward	537	1	178	19.26	9.1	−0.7511	Nucleus
*TaBZR2.6*	*TraesCS3D02G139300*	3D:98871950-98873193	Forward	1077	1	358	37.51	8	−0.5153	Nucleus
*TaBZR3.1*	*TraesCS4B02G009900*	4B:6146066-6147444	Forward	1656	7	551	62.34	5.2	−0.5284	Nucleus
*TaBZR3.2*	*TraesCS4D02G006100*	4D:3399752-3399889	Reverse	2061	8	686	75.47	5.2	−0.3704	Nucleus
*TaBZR4.1*	*TraesCS6A02G085800*	6A:54119841-54119972	Reverse	1959	6	652	73.1	6	−0.3699	Nucleus
*TaBZR4.2*	*TraesCS6A02G338000*	6A:571795527-571797497	Forward	1044	1	347	36.41	8	−0.5642	Nucleus
*TaBZR4.3*	*TraesCS6B02G116400*	6B:101917320-101917475	Forward	2019	8	672	75.48	6.5	−0.3483	Nucleus
*TaBZR4.4*	*TraesCS6B02G368700*	6B:642915742-642917800	Forward	1071	1	356	37.71	8.3	−0.6092	Nucleus
*TaBZR4.5*	*TraesCS6D02G318800*	6D:427088235-427090524	Forward	1047	1	348	36.44	7.8	−0.516	Nucleus
*TaBZR5.1*	*TraesCS7A02G354800*	7A:519131141-519132953	Forward	1080	1	359	37.84	7.5	−0.6506	Nucleus
*TaBZR5.2*	*TraesCS7B02G272900*	7B:500731158-500732708	Reverse	1080	1	359	37.86	7.5	−0.6225	Nucleus
*TaBZR5.3*	*TraesCS7D02G368000*	7D:476687645-476689882	Reverse	1080	1	359	37.8	7.5	−0.6197	Nucleus
*TaBZR6*	*TraesCSU02G078100*	Un:70283162-70283317	Forward	1959	8	652	72.95	6	−0.3601	Nucleus

ID: identity; bp: base pair; aa: amino acids; pI: isoelectric point; MW: molecular weight; KDa: Kilo dalton.

**Table 2:** The number of BZR proteins in different plant species.

Plant Species	Genome Size (Approx.)	Coding Genes	BZR Genes
*Triticum aestivum* (6n *)	17 Gb	107,891	20
*Arabidopsis thaliana* (2n)	135 MB	27,655	6
*Zea mays* (2n)	2.4 Gb	39,591	11
*Glycine max* (2n)	1.15 Gb	55,897	16
*Cajanus cajan* (2n)	833 MB	48,680	6
*Medicago sativa* (2n)	360 MB	50,444	7
*Cicer arietinum* (2n)	738 MB	25,680	6
*Solanum lycopersicum* (2n)	828 MB	34,658	9
*Beta vulgaris* (2n)	758 MB	26,521	6

* 6n = hexaploid.

## Data Availability

Data is available in the manuscript and in the Supplementary Materials.

## Supplementary Materials

The following are available online at https://www.mdpi.com/article/10.3390/ijms22168743/s1.
