# Supplementary material for: Genome-Wide Identification and Characterization of the Brassinazole-resistant (BZR) Gene Family and Its Expression in the Various Developmental Stage and Stress Conditions in Wheat (Triticum aestivum L.)
Source: Int J Mol Sci. 2021 Aug 14;22(16):8743. doi: 10.3390/ijms22168743 (PMC8395832; doi:10.3390/ijms22168743)
Supplement: Supplementary file 1 [file ijms-22-08743-s001.zip › TaBZR supplmentary figures.pdf]

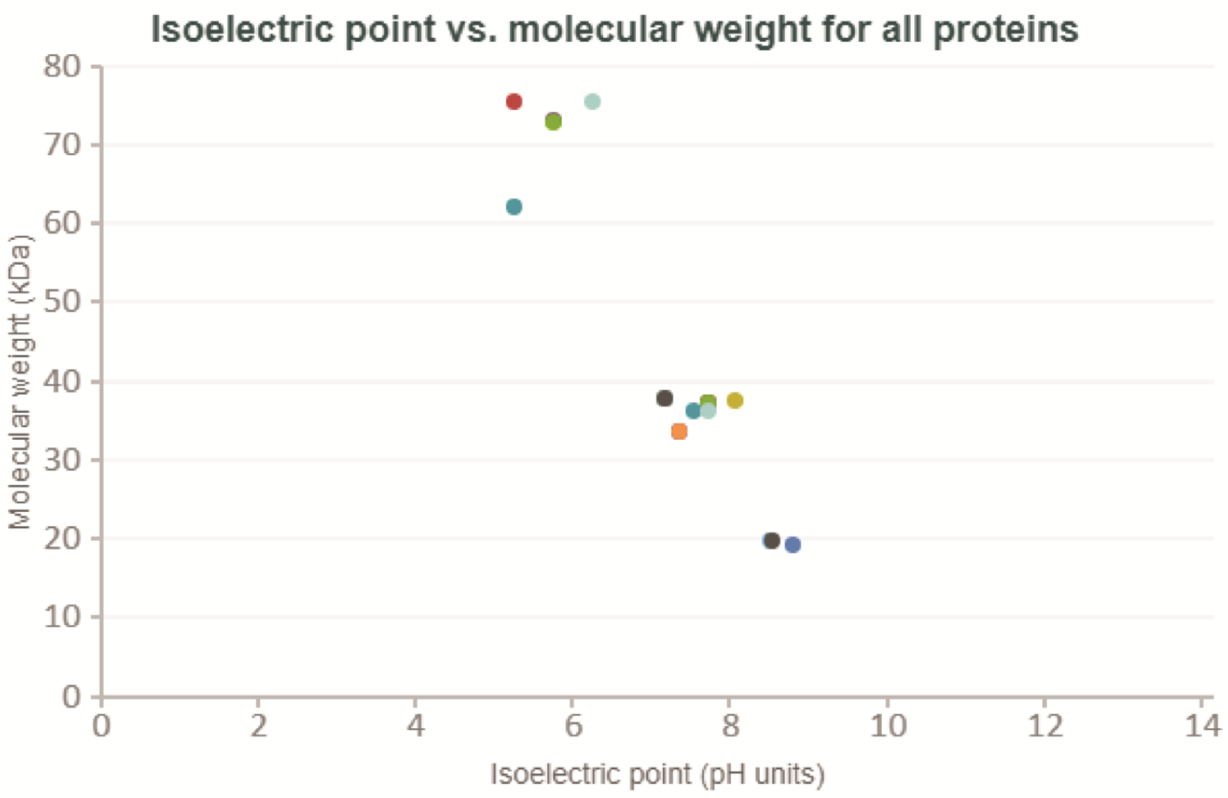

Figure S1. Molecular weight (kDa) vs. isoelectric point plots of TaBZR genes. The distinct round shape colors represent the *TaBZR* gene family members.

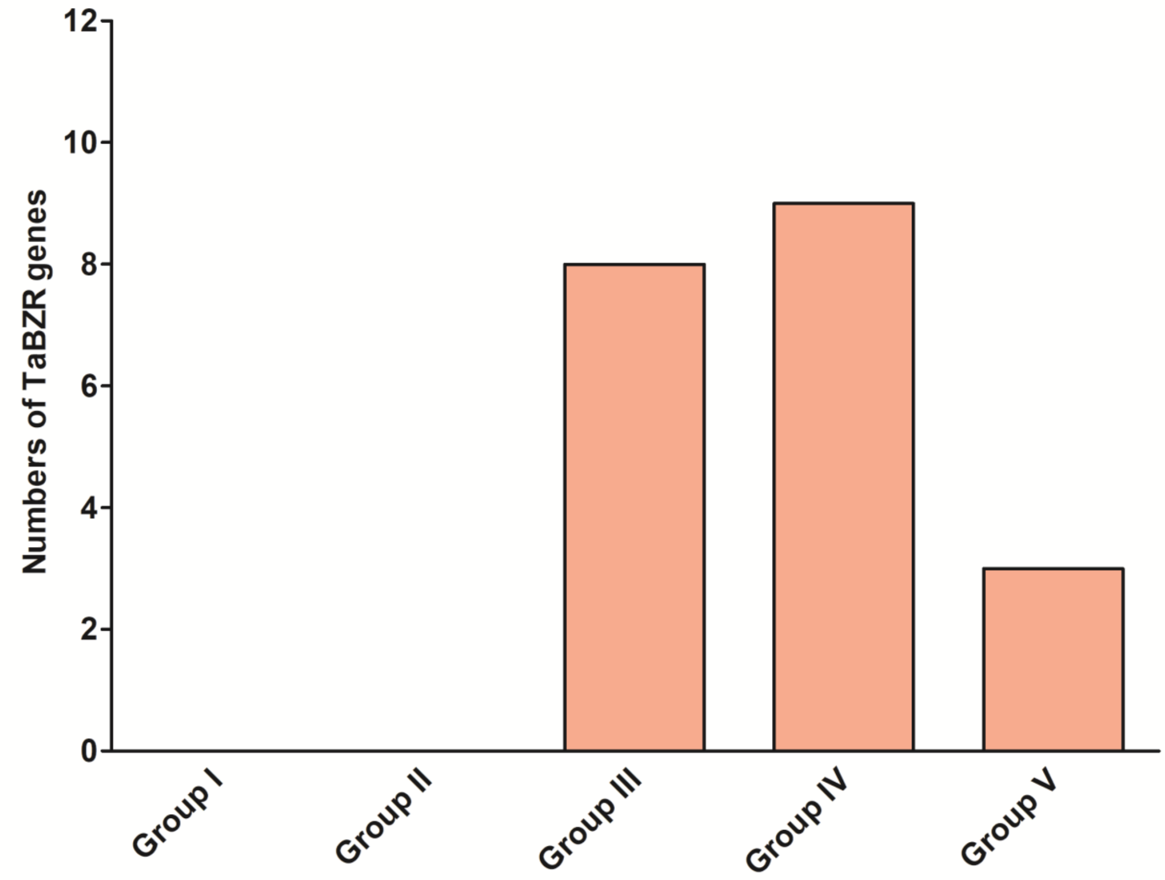

Figure S2. Distribution of *TaBZR* genes in a different group of the phylogenetic tree. The y-axis indicates the number of TaBZR genes and x-axis indicates the phylogenetic groups.

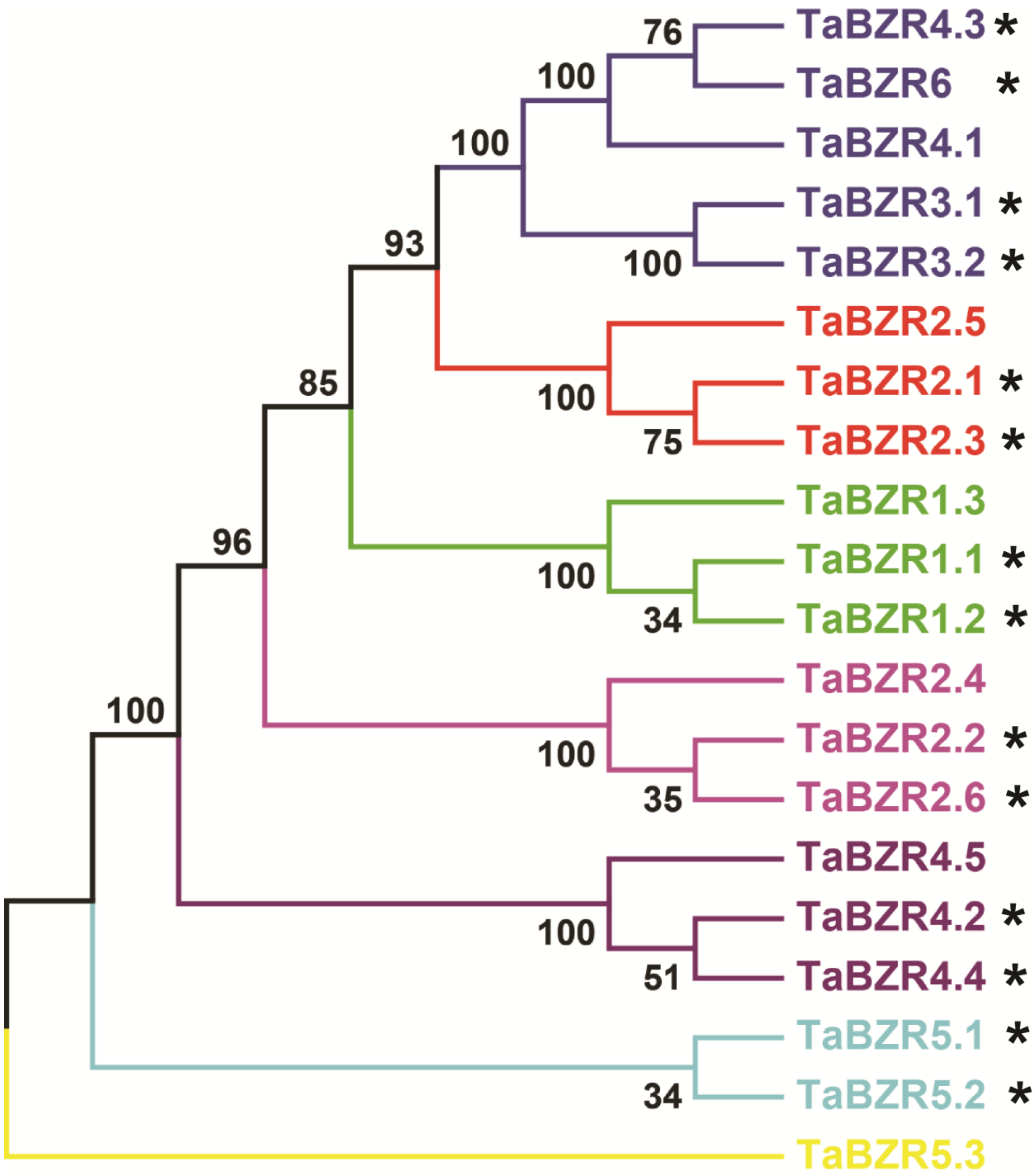

Figure S3. Phylogenetic analysis of *TaBZR* genes. A phylogenetic tree was constructed using MEGAX with the neighbor-joining (NJ) method and 1000 bootstrap replications. A black asterisk indicates the duplicated genes.

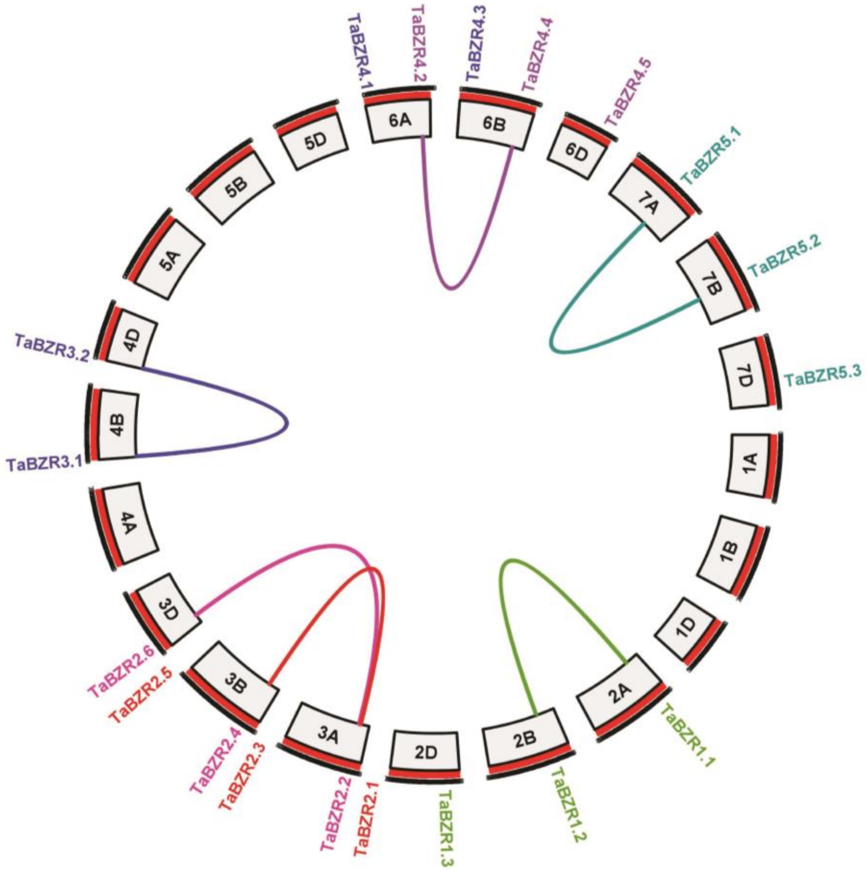

Figure S4. Chromosomal distribution and duplicated BZR gene pairs in wheat. Duplicated BZR gene pairs are connected with lines with distinct colors. The figure was generated using TB tools.

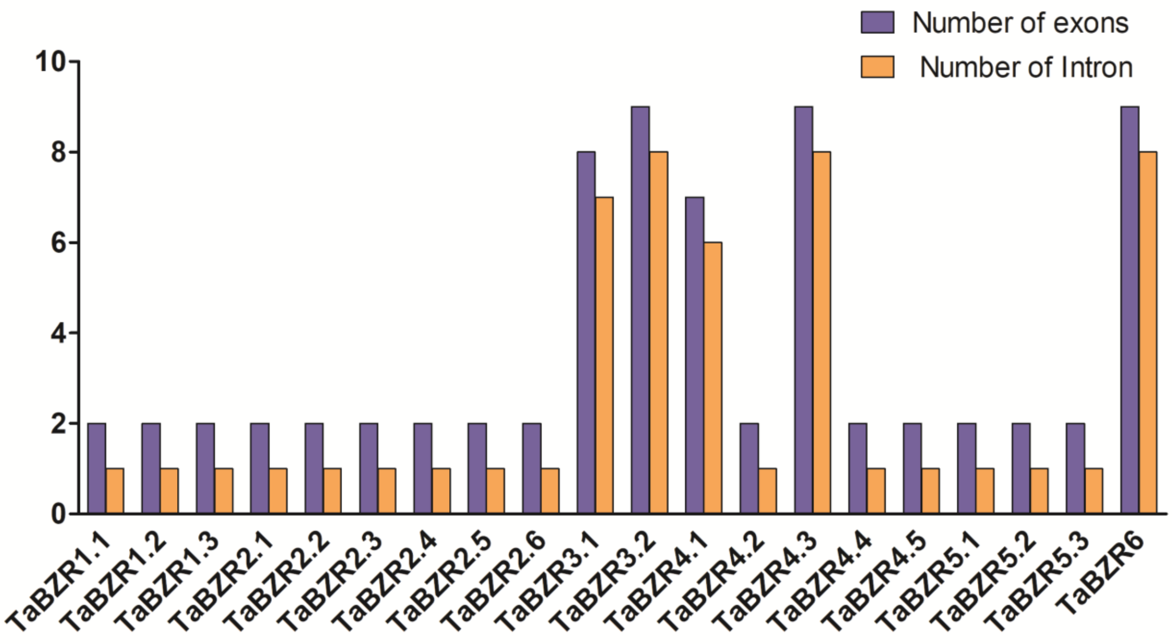

Figure S5. Distribution of exon and introns in TaBZRs gene family. The y-axis indicates the number of exon and introns and x-axis indicates the *TaBZR* genes. Exons and introns are represented by purple and orange boxes, respectively.

A

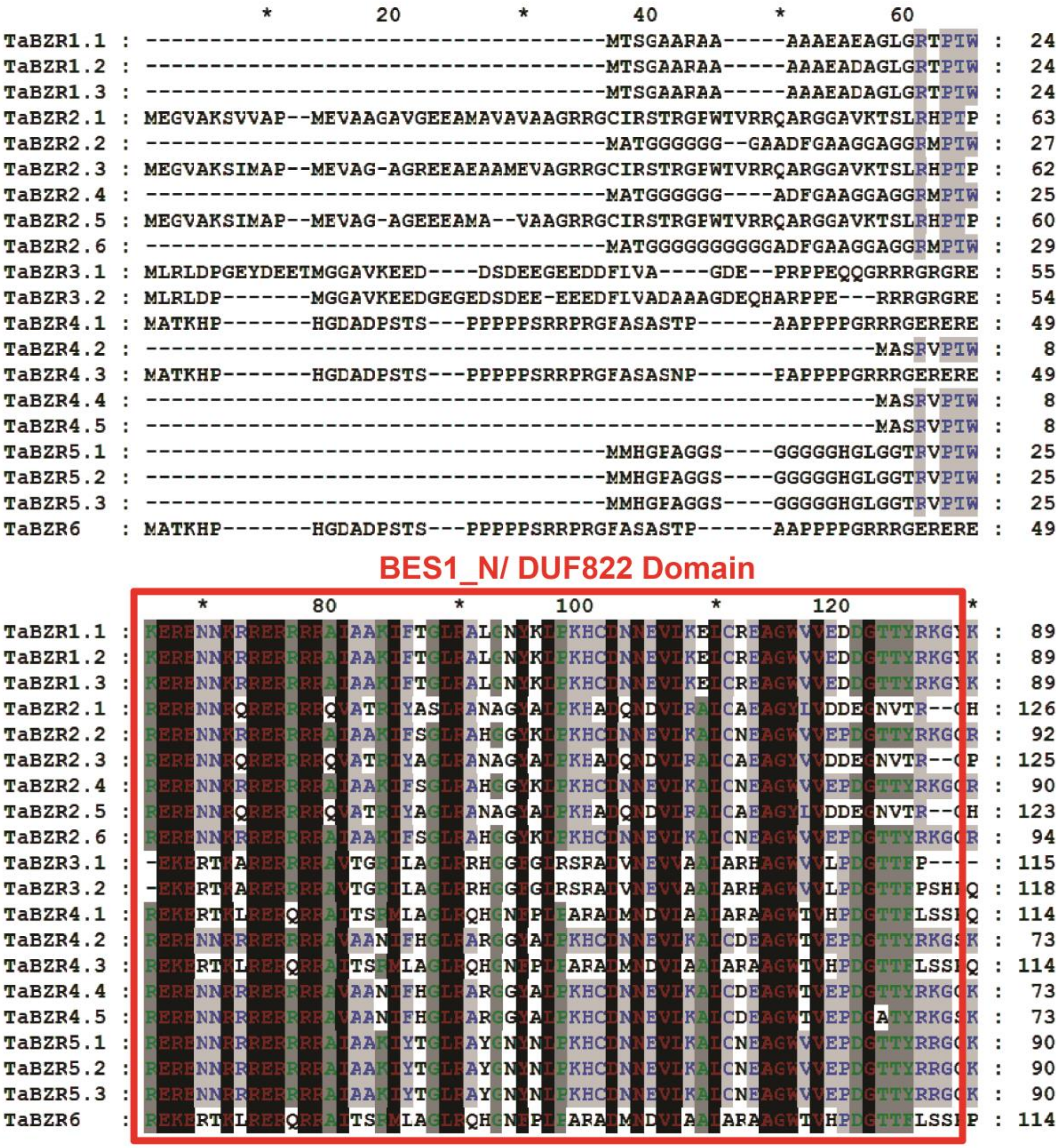
BES1_N/ DUF822 Domain
TaBZR1.1
TaBZR1.2
TaBZR1.3
TaBZR2.1
TaBZR2.2
TaBZR2.3
TaBZR2.4
TaBZR2.5
TaBZR2.6
TaBZR3.1
TaBZR3.2
TaBZR4.1
TaBZR4.2
TaBZR4.3
TaBZR4.4
TaBZR4.5
TaBZR5.1
TaBZR5.2
TaBZR5.3
TaBZR6

```
                  140         *       160         *       180         *
TaBZR1.1 : P---------------------------------------------------------------- :  90
TaBZR1.2 : P---------------------------------------------------------------- :  90
TaBZR1.3 : P---------------------------------------------------------------- :  90
TaBZR2.1 : E---------------------------------------------------------------- : 127
TaBZR2.2 : P---------------------------------------------------------------- :  93
TaBZR2.3 : E---------------------------------------------------------------- : 126
TaBZR2.4 : P---------------------------------------------------------------- :  91
TaBZR2.5 : E---------------------------------------------------------------- : 124
TaBZR2.6 : P---------------------------------------------------------------- :  95
TaBZR3.1 : ------------------------------SSSHP------------------------------ : 120
TaBZR3.2 : TPRFAMLAFAISLSSPSAAAAALPTLLFVSSSSCAPGIAVPPLAARPISRRAGCAFALRTAAASS : 183
TaBZR4.1 : PS-----LPPFAQFGAFQATSLETFVFTNSINSYAIGTPIDSQASALQTDDSLSPSSLDSVVVAE : 174
TaBZR4.2 : P---------------------------------------------------------------- :  74
TaBZR4.3 : PP-----LPPFAQFGAFQATSLETFVFTNSINSYAIGTPLDSQASALQTDDSLSPSSLDSVVVAE : 174
TaBZR4.4 : PP--------------------------------------------------------------- :  75
TaBZR4.5 : P---------------------------------------------------------------- :  74
TaBZR5.1 : P---------------------------------------------------------------- :  91
TaBZR5.2 : P---------------------------------------------------------------- :  91
TaBZR5.3 : P---------------------------------------------------------------- :  91
TaBZR6   : PP-----LPSFAQFGAFQATSLETFVFTNSINSYAIGTPLDSQASALQTDDSLSPSSLDSVVVAE : 174

             200         *       220         *       240         *       260
TaBZR1.1 : -----------------------------------------------------------PSSGPF :  96
TaBZR1.2 : -----------------------------------------------------------PSSGPF :  96
TaBZR1.3 : -----------------------------------------------------------PSSGPF :  96
TaBZR2.1 : ----------------------------------------------------------------- :   -
TaBZR2.2 : ------------------------------------------------------------AERMD :  98
TaBZR2.3 : ----------------------------------------------------------------- :   -
TaBZR2.4 : ------------------------------------------------------------AERMD :  96
TaBZR2.5 : ----------------------------------------------------------------- :   -
TaBZR2.6 : ------------------------------------------------------------AERMD : 100
TaBZR3.1 : ----------------------------------------------------------------- :   -
TaBZR3.2 : PFAVSRAVPEGGFASPLLAVFVPDDEDADAAMDGGDGGEQTGLATRFAVAPPRPPPERDFAGTPY : 248
TaBZR4.1 : QSIKNESYGNSSSANSINCMGSDQLLRASAVWAG------------------------DFTKTPY : 215
TaBZR4.2 : ----------------------------------------------------------VTAEGTN :  81
TaBZR4.3 : QSIKNESYGNSSSANSINCMGSDQLLRASAVWAG------------------------DFTKTPY : 215
TaBZR4.4 : ----------------------------------------------------------AAAEGAN :  82
TaBZR4.5 : ----------------------------------------------------------LAAEGAN :  81
TaBZR5.1 : -----------------------------------------------------------PPQARP :  97
TaBZR5.2 : -----------------------------------------------------------PPQVRP :  97
TaBZR5.3 : -----------------------------------------------------------PPQART :  97
TaBZR6   : QSIKNESYGNSSSANSINCMGSDQLLRASAVCAG------------------------DFTKTPY : 215
```

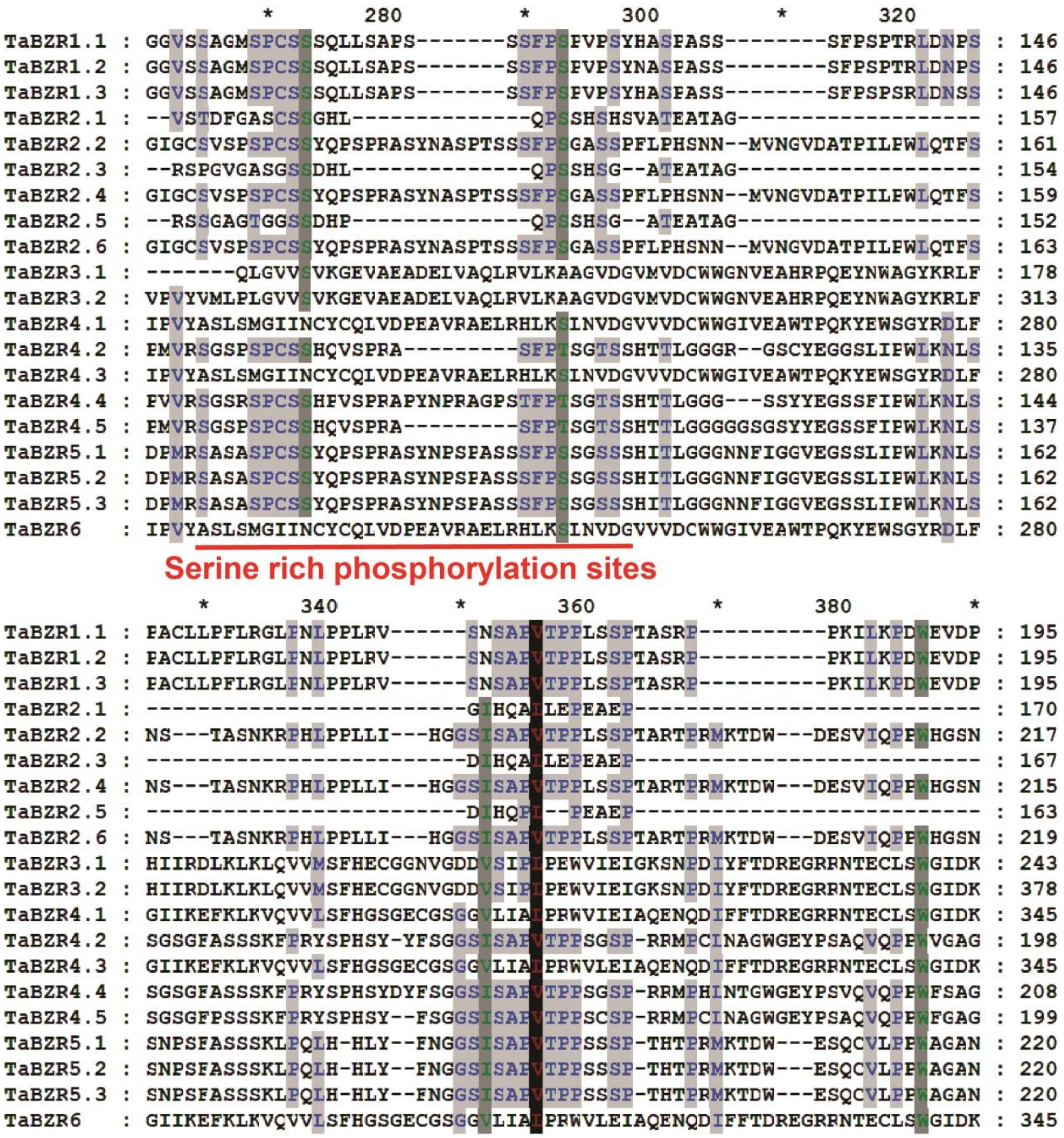
Serine rich phosphorylation sites

```
                       400         *        420         *        440         *
TaBZR1.1 : FR----HPFFALSAFASPTRGRRHEHPDTIPECDESDVSTVDSGRWISFQMATT----------A : 246
TaBZR1.2 : FR----HPFFALSAFASPTRGRRHEHPDTIPECDESDVSTVDSGRWISFQMATT----------A : 246
TaBZR1.3 : FR----HPFFALSAFASPTRGRRHEHPDTIPECDESDVSTVDSGRWISFQMATT----------A : 246
TaBZR2.1 : ---------------------------------KHKISLELTLSFSYM----------------- : 185
TaBZR2.2 : ------SPCVVNSTPPSP--G-RQMVPDP-AWIAGIQISSTSPSSPTFSLMSSNP--FSVFKEAI : 270
TaBZR2.3 : ---------------------------------KHKISLELTLSFSYM----------------- : 182
TaBZR2.4 : ------SPCVVNSTPPSP--G-RQMVPDP-AWIAGIQISSTSPSSPTFSLMSSNP--FSVFKEAI : 268
TaBZR2.5 : ---------------------------------KHKISLELTLSFTYM----------------- : 178
TaBZR2.6 : ------SPCVVNSTPPSP--G-RQMVPDP-AWIAGIQISSTSPSSPTFSLMSSNP--FSVFKEAI : 272
TaBZR3.1 : ERVLQGRTAVEVYFDFMRSFRVEFDEYFEDGIISEIEVGLGACGELRYPSYAANHGWKYPGIGEF : 308
TaBZR3.2 : ERVLQGRTAVEVYFDFMRSFRVEFDEYFEDGIISEIEVGLGACGELRYPSYAANHGWKYPGIGEF : 443
TaBZR4.1 : ERVLRGRTGIEVYFDFMRSFHMEFRSLSEEGLISAIEVGLGASGELRYPSCPEKMGWRYPGIGEF : 410
TaBZR4.2 : GSGYDDYASLFNSTPSSPRGG-QGVGPDFAAWLSGFQISSAGPSSPTYSLMAPSNPFRVSGEMAA : 262
TaBZR4.3 : ERVLRGRTGIEVYFDFMRSFHMEFRSLSEEGLISAIEVGLGASGELRYPSCPEKMGWRYPGIGEF : 410
TaBZR4.4 : GSAYDDYASLFNSTPSSPRGG-HGVGPDP-AWLSGFQISSAGPSSPTYSLMAPSNPFRVSGEMAA : 271
TaBZR4.5 : GSGYDDYASLFNSTPSSPRGGSHGVGPDP-AWLSGFQISSAGPSSPTYSLMAPSSSFRVFGEMAA : 263
TaBZR5.1 : ------YTSLFNSTPPSP--G-HHVAPDP-AWIAGFQISSAGPSSPTYNLVSHN-------PFGI : 268
TaBZR5.2 : ------YTSLFNSTPPSP--G-HHVAPDP-AWIAGFQISSAGPSSPTYNLVSHN-------PFGI : 268
TaBZR5.3 : ------YASLFNSTPPSP--G-HHVAPDP-AWIAGFQISSAGPSSPTYNLVSHN-------PFGI : 268
TaBZR6   : ERVLRGRTGIEVYFDFMRSFHMEFRSLSEEGLISAIEVGLGASGELRYPSCPEKMGWRYPGIGEF : 410

                 460         *        480         *        500         *        520
TaBZR1.1 : PTSFAYNLVNLGASSSNSMEMEG--------MAGERGRSGPEEEFDKG----------------- : 286
TaBZR1.2 : PTSFAYNLVNLGASSSNSMELDG--------MAGERGRSGPEEEFDKG----------------- : 286
TaBZR1.3 : PTSFAYNLVNLGASSSNSMEMDG--------MAGERGRSGPEEEFDKG----------------- : 286
TaBZR2.1 : ----------------------------------------------------------------- :   -
TaBZR2.2 : PGGGSSRMCTPGQSGTCSFVIPG-MARHPDVHMMDV--VSDEFAFGSSTNGGA------------ : 320
TaBZR2.3 : ----------------------------------------------------------------- :   -
TaBZR2.4 : PGGGSSRMCTPGQSGTCSFVIPG-MARHPDVHMMDV--VSDEFAFGSSTNGGA------------ : 318
TaBZR2.5 : ----------------------------------------------------------------- :   -
TaBZR2.6 : PGGGSSRMCTPGQSGTCSFVIPG-MARHPDVHMMDV--VSDEFAFGSSTNGGA------------ : 322
TaBZR3.1 : QCYDRYLQKNLRFAAEARGHAMWAKSPDNAGHYNSEFNNTGFFCDGGDYDSYYGRFFINWYAQVL : 373
TaBZR3.2 : QCYDRYLQKNLRFAAEARGHAMWAKSPDNAGHYNSEFNNTGFFCDGGDYDSYYGRFFINWYAQVL : 508
TaBZR4.1 : QCYDRYMQKNLRQSALTRGHLFWARGPDNAGYYNSRSHETGFFCDGGDYDSYYGRFFINWYSGIL : 475
TaBZR4.2 : ATASSSRMRTPCQSGTCSFVA---IAVHGDVQMGAR--AEDDFEFGSG----------------- : 305
TaBZR4.3 : QCYDRYMQKNLRQSALTRGHLFWARGPDNAGYYNSRSHETGFFCDGGDYDSYYGRFFINWYSGIL : 475
TaBZR4.4 : ATASSSRMRTPCQSGTCSFVA---IAAHGDVQMEHR--EADDFAFGSG----------------- : 314
TaBZR4.5 : ATAGSSRMCTFAQSGTCSFVM---IAVHGDVQMGAR--AEDDFEFGSG----------------- : 306
TaBZR5.1 : AIASSSRACTPGQSGTCSFVMGDHAFAHHDVQMEMADGAPDDFAFGSNSNG-------------- : 319
TaBZR5.2 : AIASSSRACTPGQSGTCSFVMGDHAFAHHDVQMEMVEGAADDFAFGSNSNG-------------- : 319
TaBZR5.3 : AIASSSRVCTPGQSGTCSFAMGDHAFAHHDVQMEMVEGAADDFAFGSNSNG-------------- : 319
TaBZR6   : QCYDRYMQKNLRQSALARGHLFWARGPDNAGYYNSRSHETGFFCDGGDYDSYYGRFFINWYSGIL : 475
```

```
                             *       540         *       560         *       580
TaBZR1.1 : ----------RVTPWEGERIHEVA-------AEE---LELTLGVGSK------------------ : 313
TaBZR1.2 : ----------RVTPWEGERIHEVA-------AEE---LELTLGVGSK------------------ : 313
TaBZR1.3 : ----------RVTPWEGERIHEVA-------AEE---LELTLGVGSK------------------ : 313
TaBZR2.1 : ----------------------------------------------------------------- :   -
TaBZR2.2 : ----QQATAGLVRAWEGERIHEDS-------GSD--ELELTL--GSSRTRS-------------- : 356
TaBZR2.3 : ----------------------------------------------------------------- :   -
TaBZR2.4 : ----QQATAGLVRAWEGERIHEDS-------GSD--ELELTL--GSSRTRS-------------- : 354
TaBZR2.5 : ----------------------------------------------------------------- :   -
TaBZR2.6 : ----QQATAGLVRAWEGERIHEDS-------GSD--ELELTL--GSSRTRS-------------- : 358
TaBZR3.1 : LDHADRVIMLARLAFEGSAIAVKVSGIHWWYKIASHAAELTAGFYNPCNRDGYAPIAQVLKKHGA : 438
TaBZR3.2 : LDHADRVIMLARLAFEGSAIAVKVSGIHWWYKIASHAAELTAGFYNPCNRDGYAPIAQVLKKHGA : 573
TaBZR4.1 : IDHVDQVLSLATLAFDGAAIVVKIPSIYWWYRIASHAAELTAGFYNPTNRDGYSPVFRMLKKHSI : 540
TaBZR4.2 : ------GRPVSVNAWEGERIHEVC-------ASDEPELDLTLGLGSSNTRASALN---------- : 347
TaBZR4.3 : IDHVDQVLSLATLAFDGAAIVVKIPSIYWWYRIASHAAELTAGFYNPTNRDGYSPVFRMLKKHSI : 540
TaBZR4.4 : ------GRPVLVNAWEGERIHEVC-------ASDEHELDLTLGLGSSKTRAGAVN---------- : 356
TaBZR4.5 : ------GRPVSVNAWEGERIHEVC-------ASDEPELELTLGLGSSKTRAGAVN---------- : 348
TaBZR5.1 : ----NNGSPGLVKAWEGERIHEEC-------ASD--ELELTL--GSSRTRGEQPF---------- : 359
TaBZR5.2 : ----NNGSPGLVKAWEGERIHEEC-------ASD--ELELTL--GSSRTRGEPPF---------- : 359
TaBZR5.3 : ----NNGSPGLVKAWEGERIHEEC-------ASD--ELELTL--GSSRTRGEPPF---------- : 359
TaBZR6   : IDHVDQVLSLATLAFDGAAIVVKIPSIYWWYRIASHAAELTAGFYNPTNRDGYSPVFRMLKKHSI : 540
```

C-terminal domain

```
                         *       600         *       620         *       640         *
TaBZR1.1 : ----------------------------------------------------------------- :   -
TaBZR1.2 : ----------------------------------------------------------------- :   -
TaBZR1.3 : ----------------------------------------------------------------- :   -
TaBZR2.1 : ----------------------------------------------------------------- :   -
TaBZR2.2 : ----------------------------------------------------------------- :   -
TaBZR2.3 : ----------------------------------------------------------------- :   -
TaBZR2.4 : ----------------------------------------------------------------- :   -
TaBZR2.5 : ----------------------------------------------------------------- :   -
TaBZR2.6 : ----------------------------------------------------------------- :   -
TaBZR3.1 : ALNFTCVELRTMDQHEVYPEALADPEGIVWQVINAAWDAGIQVASENALPCYDRDGFNKTLENAK : 503
TaBZR3.2 : ALNFTCVELRTMDQHEVYPEALADPEGIVWQVINAAWDAGIQVASENALPCYDRDGFNKTLENAK : 638
TaBZR4.1 : ILKVVCYGPEFTVQEND--EAFADPEGLTWQVMNAAWDHGLSVSVESALPCLDVDMYSRILDTAK : 603
TaBZR4.2 : ----------------------------------------------------------------- :   -
TaBZR4.3 : ILKVVCYGPEFTVQENG--EAFADPEGLTWQVMNAAWDHGLSVSVESSLPCLDVDMYSRILDTAK : 603
TaBZR4.4 : ----------------------------------------------------------------- :   -
TaBZR4.5 : ----------------------------------------------------------------- :   -
TaBZR5.1 : ----------------------------------------------------------------- :   -
TaBZR5.2 : ----------------------------------------------------------------- :   -
TaBZR5.3 : ----------------------------------------------------------------- :   -
TaBZR6   : ILKVVCYGPEFTVQEND--EAFADPEGLTWQVMNAAWDHGLSVSVESALPCLDVDMYSRILDTAK : 603
```

```
                    660         *        680         *        700         *
TaBZR1.1 : ----------------------------------------------------------------- :   -
TaBZR1.2 : ----------------------------------------------------------------- :   -
TaBZR1.3 : ----------------------------------------------------------------- :   -
TaBZR2.1 : ----------------------------------------------------------------- :   -
TaBZR2.2 : ----------------------------------------------------------------- :   -
TaBZR2.3 : ----------------------------------------------------------------- :   -
TaBZR2.4 : ----------------------------------------------------------------- :   -
TaBZR2.5 : ----------------------------------------------------------------- :   -
TaBZR2.6 : ----------------------------------------------------------------- :   -
TaBZR3.1 : PRNDPDGRHLFGFTYLRLCSTLFE-GPNLPEFERFVKRMHGEAVHDLRA---------------- : 551
TaBZR3.2 : PRNDPDGRHLFGFTYLRLCSTLFE-GPNLPEFERFVKRMHGEAVHDLRA---------------- : 686
TaBZR4.1 : PRNDPDRHHLSFFAYRQRTPFLLQRDVCFSELETFVKCMHGEATQNFVD---------------- : 652
TaBZR4.2 : ----------------------------------------------------------------- :   -
TaBZR4.3 : PRNDPDRHHLSFFAYRQRTPFLLQRDACFSELETFVKCMHELCRLTYCSLRSANFRIPFVAIARL : 668
TaBZR4.4 : ----------------------------------------------------------------- :   -
TaBZR4.5 : ----------------------------------------------------------------- :   -
TaBZR5.1 : ----------------------------------------------------------------- :   -
TaBZR5.2 : ----------------------------------------------------------------- :   -
TaBZR5.3 : ----------------------------------------------------------------- :   -
TaBZR6   : PRNDPDRHHLSFFAYRQRTPFLLQRDVCFSELETFVKCMHGEATQNFVD---------------- : 652
```

```
TaBZR1.1 : ---- :   -
TaBZR1.2 : ---- :   -
TaBZR1.3 : ---- :   -
TaBZR2.1 : ---- :   -
TaBZR2.2 : ---- :   -
TaBZR2.3 : ---- :   -
TaBZR2.4 : ---- :   -
TaBZR2.5 : ---- :   -
TaBZR2.6 : ---- :   -
TaBZR3.1 : ---- :   -
TaBZR3.2 : ---- :   -
TaBZR4.1 : ---- :   -
TaBZR4.2 : ---- :   -
TaBZR4.3 : NSYC : 672
TaBZR4.4 : ---- :   -
TaBZR4.5 : ---- :   -
TaBZR5.1 : ---- :   -
TaBZR5.2 : ---- :   -
TaBZR5.3 : ---- :   -
TaBZR6   : ---- :   -
```

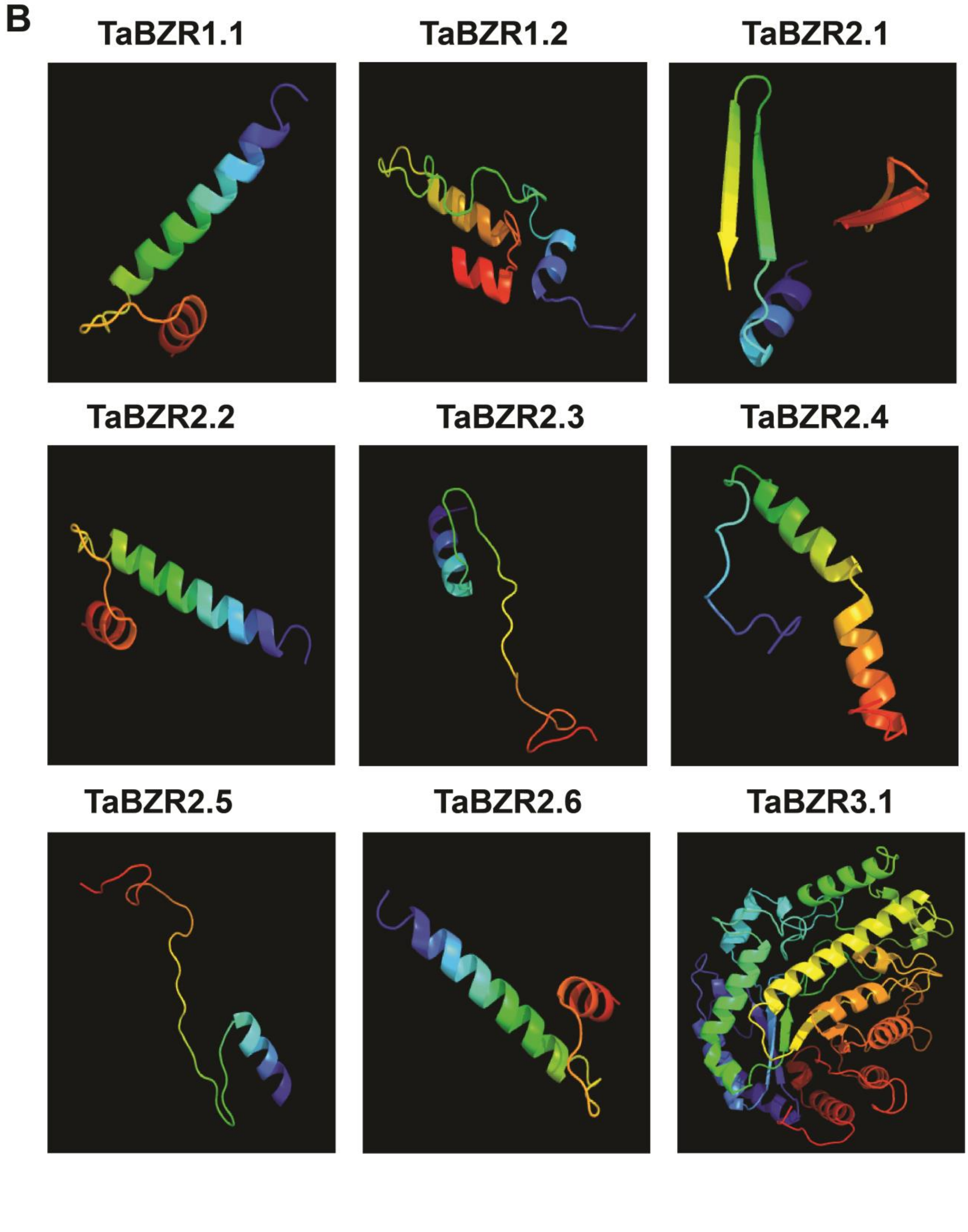
B
TaBZR1.1
TaBZR1.2
TaBZR2.1
TaBZR2.2
TaBZR2.3
TaBZR2.4
TaBZR2.5
TaBZR2.6
TaBZR3.1

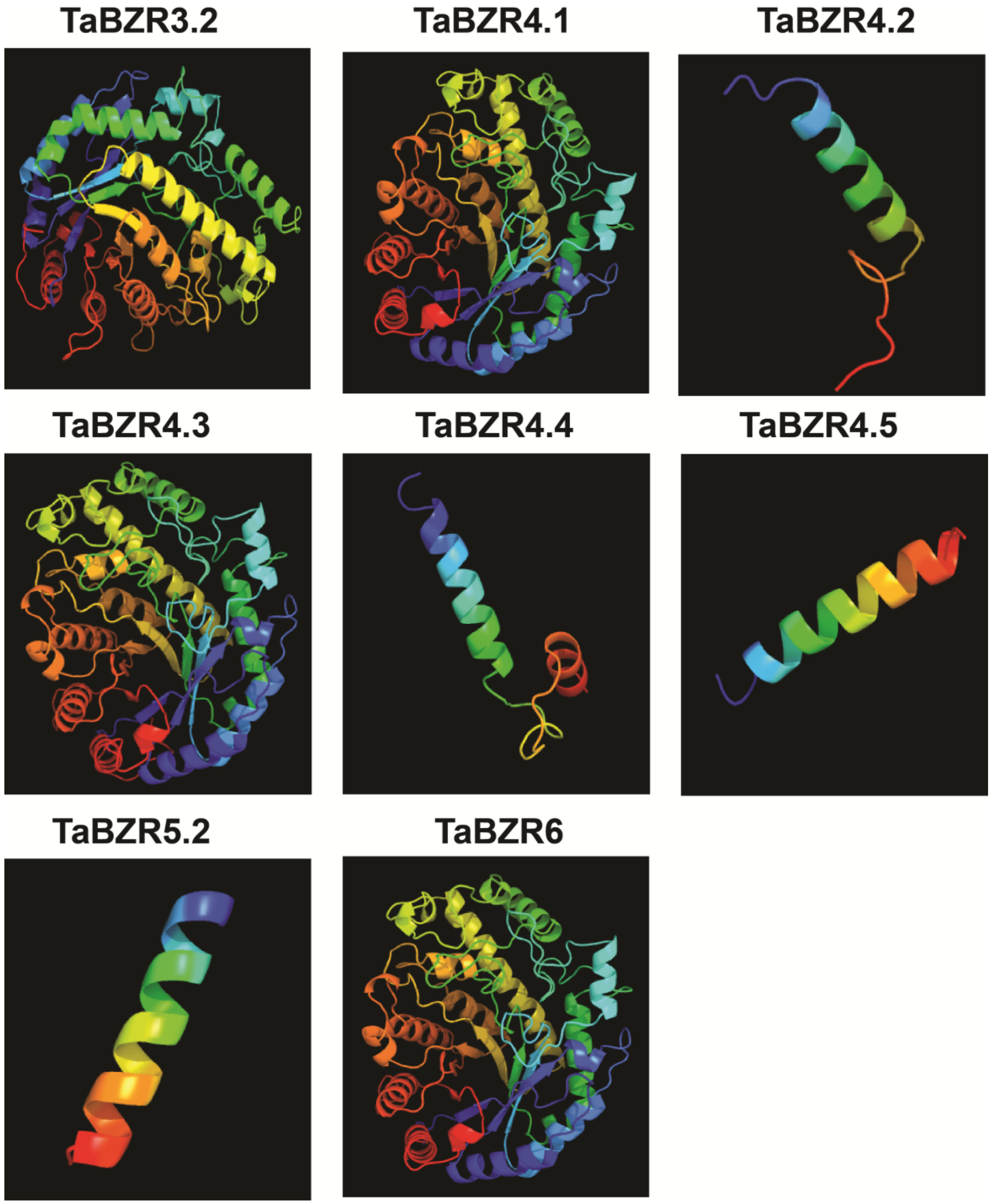

Figure S6. Alignment and 3-dimensional structure of the TaBZR protein sequences. A. The conserved BES_N1/DUF822 domain is boxed with red color. Red lines indicate serine-rich phosphorylation sites and the C-terminal domain. Colored and shaded amino acids are chemically similar residues. Dashes indicate gaps introduced to maximize the alignment of the homologous region. **B.** Predicted 3D structures TaBZR proteins.

A

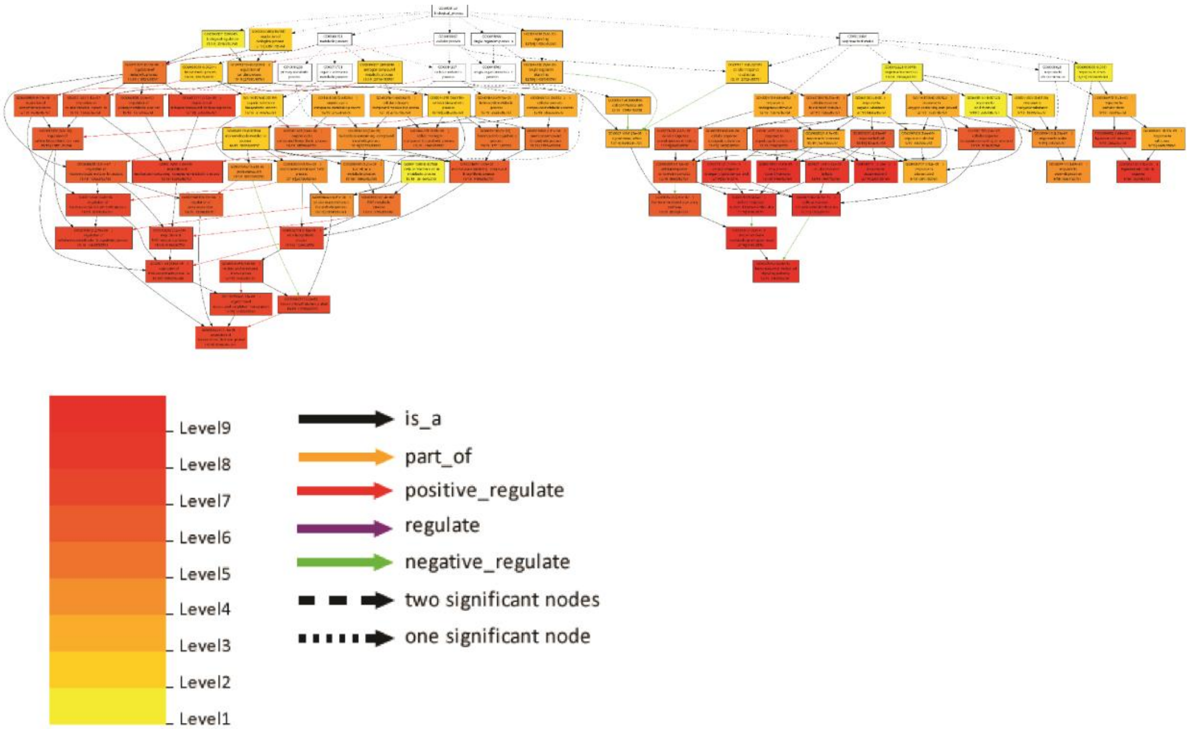
Level9
Level8
Level7
Level6
Level5
Level4
Level3
Level2
Level1
is_a
part_of
positive_regulate
regulate
negative_regulate
two significant nodes
one significant node

B

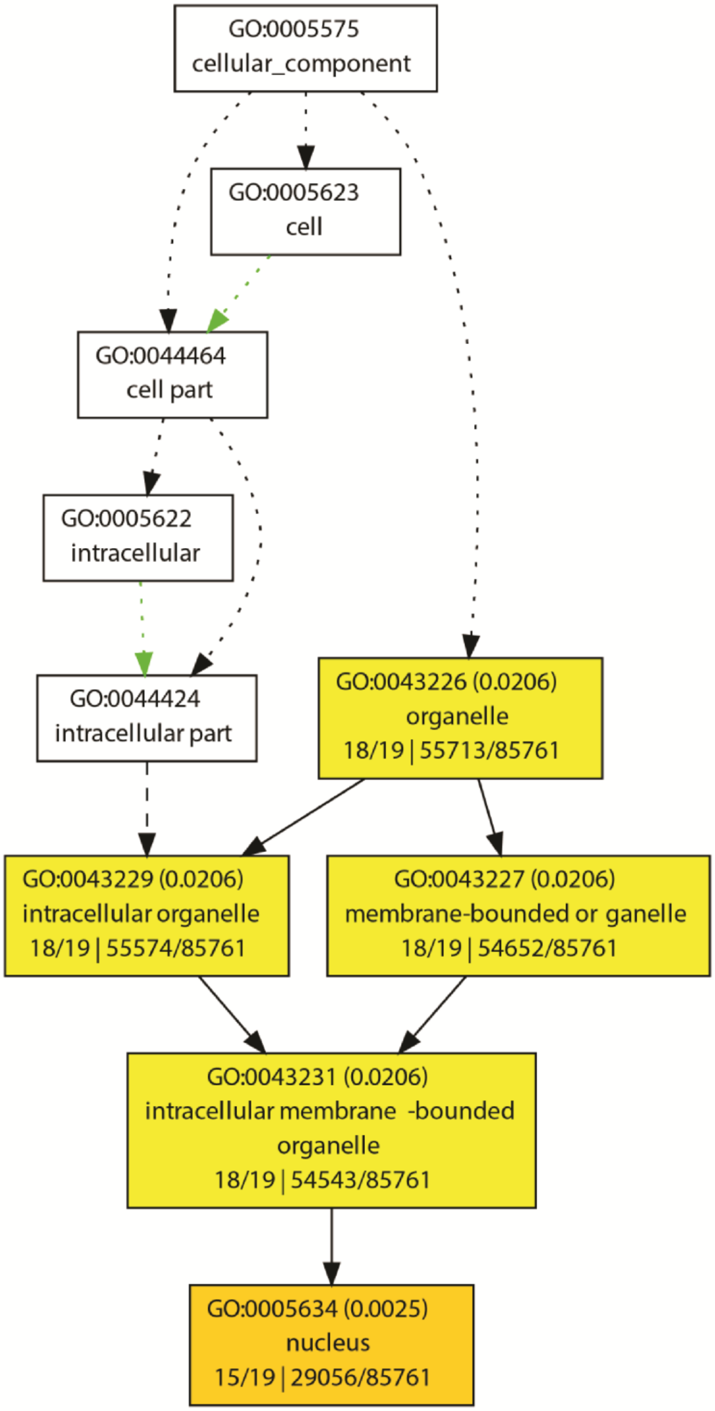

GO:0005575
cellular_component
GO:0005623
cell
GO:0044464
cell part
GO:0005622
intracellular
GO:0044424
intracellular part
GO:0043226 (0.0206)
organelle
18/19 | 55713/85761
GO:0043229 (0.0206)
intracellular organelle
18/19 | 55574/85761
GO:0043227 (0.0206)
membrane-bounded or ganelle
18/19 | 54652/85761
GO:0043231 (0.0206)
intracellular membrane -bounded
organelle
18/19 | 54543/85761
GO:0005634 (0.0025)
nucleus
15/19 | 29056/85761

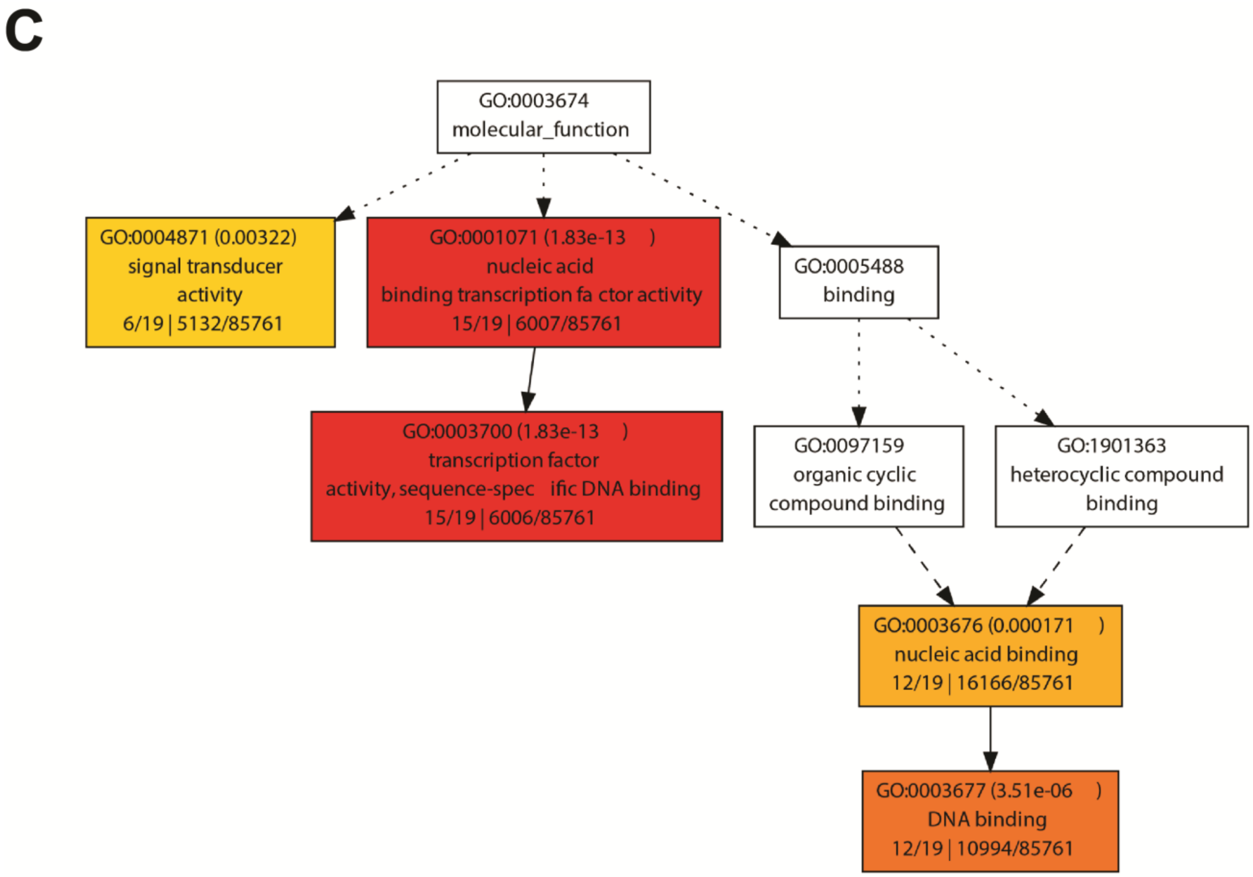

Figure S7. Gene ontology term distribution *TaBZR* gene family predicted using AgriGO A. Biological Process. **B**. Cellular component. **C.** Molecular function.

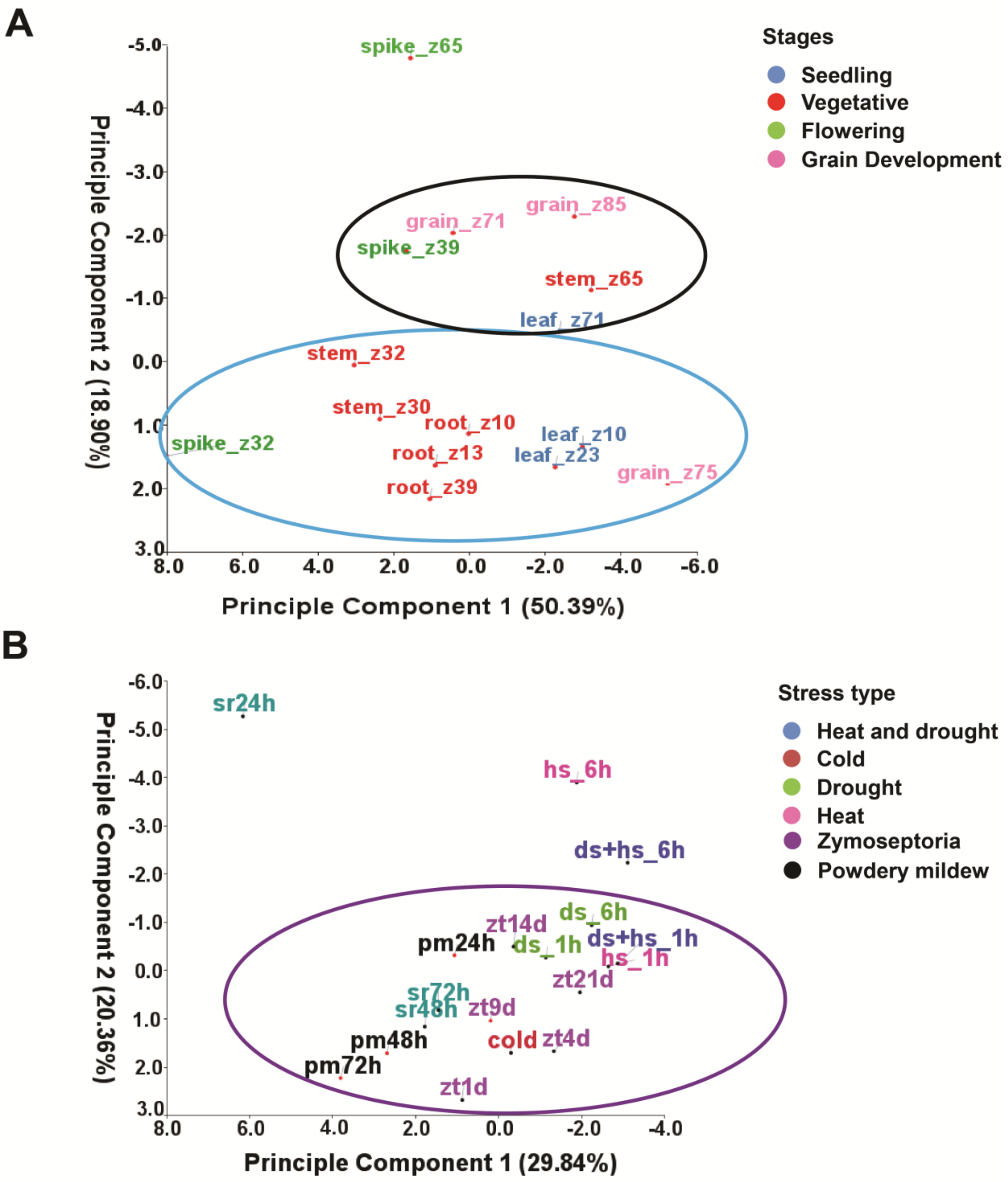

Figure S8. PCA plots displaying grouping of different A. Developmental stages **B.** Biotic and abiotic stress conditions based on the *TaBZR* expression pattern. DS: Drought stress, HS: Heat stress, Zt: *Zymoseptoria tritici*, PM: Powdery mildew; SR: Stripe rust, h: hour and d: days.
